# Molecular Diversity and Evolutionary Relatedness of Paulownia Witches’-Broom Phytoplasma in Different Geographical Distributions in China

**DOI:** 10.3390/biology11111611

**Published:** 2022-11-03

**Authors:** De-Zhi Kong, Cai-Li Lin, Shao-Shuai Yu, Guo-Zhong Tian, Hai-Bin Ma, Sheng-Jie Wang

**Affiliations:** 1 Key Laboratory of Forest Protection of National Forestry and Grassland Administration, Ecology and Nature Conservation Institute, Chinese Academy of Forestry, Beijing 100091, China; 2 Coconut Research Institute, Chinese Academy of Tropical Agricultural Sciences, Wenchang 571339, China; 3 The Key Laboratory of National Forestry and Grassland Administration for Tropical Forestry Research, Research Institute of Tropical Forestry, Chinese Academy of Forestry, Guangzhou 510520, China

**Keywords:** paulownia witches’-broom (PaWB), multilocus sequence typing (MLST), phytoplasma, phylogeny, genetic diversity

## Abstract

**Simple Summary:**

Paulownia witches’-broom (PaWB) is a severe and widespread disease caused by an obligate pathogen phytoplasma that causes substantial economic losses and ecological damage in China. As such, in this study, we first obtained detailed insight into the molecular diversity of PaWB phytoplasmas populations from different geographical distributions with multilocus sequence typing (MLST). There was comparatively high genetic diversity among the PaWB strains. Mutations in *tuf* and *rpoB* were driven by some positive selection pressure in the PaWB phytoplasma populations, and nine housekeeping genes, except for *tuf*, followed the neutral evolutionary model. However, the recombination among PaWB phytoplasma sequence types (STs) of each housekeeping gene, except for *dnaK*, was absent. The results of phylogenetic analysis revealed that the strains were clustered into two representative lineages of PaWB phytoplasma with obvious geographical differentiation. The predominant ST1 strains of CC1 were distributed in four geographical populations. The special PaWB phytoplasma CC2 was mainly distributed in Jiangxi and Fujian provinces in the south of the Yangtze River. The ST diversity of the northwest China population was the highest, including a singleton from Xi’an Shaanxi province, which was closely related with CC2.

**Abstract:**

To reveal the distribution and transmission pathway of Paulownia witches’-broom (PaWB) disease, which is caused by phytoplasmas related to genetic variation, and the adaptability to the hosts and environments of the pathogenic population in different geographical regions in China, in this study, we used ten housekeeping gene fragments, including *rp*, *fusA*, *secY*, *tuf*, *secA*, *dnaK*, *rpoB*, *pyrG*, *gyrB*, and *ipt*, for multilocus sequence typing (MLST). A total of 142 PaWB phytoplasma strains were collected from 18 provinces or municipalities. The results showed that the genetic diversity was comparatively higher among the PaWB phytoplasma strains, and substantially different from that of the other 16SrI subgroup strains. The number of gene variation sites for different housekeeping genes in the PaWB phytoplasma strains ranged from 1 to 14 SNPs. Among them, *rpoB* (1.47%) and *dnaK* (1.12%) had higher genetic variation, and *rp* (0.20%) had the least genetic variation. The *tuf* and *rpoB* genes showed the fixation of positively selected beneficial mutations in the PaWB phytoplasma populations, and all housekeeping genes except *tuf* followed the neutral evolutionary model. We found an absence of recombination among PaWB phytoplasma sequence types (STs) for each housekeeping gene except *dnaK*, and no evidence for such recombination events for concatenated sequences of PaWB phytoplasma strains. The 22 sequence types were identified among the concatenated sequences of seven housekeeping genes (*rp*, *fusA*, *secY*, *secA*, *tuf*, *dnaK,* and *rpoB*) from 105 representative strains. We analyzed all 22 STs by goeBURST algorithm, forming two clonal complexes (CCs) and three singletons. Among them, ST1, as the primary founder of CC1, had the widest geographical distribution, accounting for 72.38% of all strains, with a high frequency of shared sequence type. The results of phylogenetic analysis of the concatenated sequences further revealed that the 105 strains were clustered into two representative lineages of PaWB phytoplasma, with obvious geographical differentiation. The ST1 strains of highly homogeneous lineage-1 were a widespread and predominant population in diseased areas. Lineage-2 contained strains from Jiangxi, Fujian, and Shaanxi provinces, highlighting the close genetic relatedness of the strains in these regions, which was also consistent with the results of most single-gene phylogenetic analysis of each gene. We also found that the variability in the northwest China population was higher than in other geographical populations; the range of genetic differentiation between the south of the Yangtze River population and the Huang-huai-hai Plain (or southwest China) population was relatively large. The achieved diversity and evolution data, as well as the MLST technique, are helpful for epidemiological studies and guiding PaWB disease control decisions.

## 1. Introduction

Paulownias are deciduous trees belonging to *Paulownia* in the Paulowniaceae [1], which are distributed in most parts in China. It has been cultivated and used for more than 2000 years in China. Some species have been introduced to many countries, such as Vietnam, Japan, South Korea, Brazil, and Australia. Paulownias are a well-liked option for enhancing the ecological environment and mitigating the effects of climate change because they have an excellent capacity for sequestering carbon and a remarkable tolerance to salinity and drought. Considering that, they may play a role in China’s policies for achieving carbon neutrality and peaking carbon dioxide emissions.

PaWB is a severe and widespread disease caused by phytoplasmas infecting the phloem sieve tubes of paulownias. At present, the main natural host of PaWB phytoplasma is paulownias. The disease is transmitted by vegetative propagation materials, such as the roots and seedlings of infected paulownias, and by vector insects, such as the stink bug, *Halyomorpha halys* (Stål) [2]. It often causes a large number of axillary buds, shortened internodes, floral metamorphosis, leaf chlorosis, and other symptoms in host plants. PaWB presently occurs in the major paulownia planting areas in China, Japan, and South Korea. After the 1960s, with large-scale paulownia planting and the absence of efficient pathogen detection and prophylactic control techniques in China, the occurrence of and harm caused by PaWB gradually increased. A current survey showed that the incidence of PaWB disease in severely affected areas can reach more than 70% (e.g., Jiaocheng, Shanxi) [3]. The historic findings of phytoplasmas, a pathogenic mycoplasma-like organism (MLO) in the sieve tube of the phloem of infected paulownia, mulberry, potato, etc., in Japan by Doi et al. [4] in 1967 resulted in substantial progress in studies on PaWB disease. Currently, many study data on the various facets of this disease have already been accumulated, ranging from disease survey and diagnosis, pathogen identification, pathogenicity, vegetative and insect vector transmission pathways, epidemiology, and control strategies [5,6,7]. The application of molecular techniques in the phytoplasma field has solved many key problems involving phytoplasma detection, identification, and classification, and has facilitated disease management [8]. Based on 16S rRNA gene data, PaWB phytoplasma is assigned to the aster yellows group, subgroup D (16SrI-D), which is one of the ‘*Candidatus Phytoplasma asteris*’-related strains [9,10,11,12]. Although conventional classification evidence based on 16S rRNA and several other conservative genes has supported its taxonomic status, we have long wondered whether a member of the PaWB phytoplasma exists that has a narrow host range and with a specified insect vector within this subgroup and how this member differs from the reference strain (Aster yellows witches’-broom (AYWB) or ‘*Ca. P. asteris*’).

The ability to accurately identify the strains of infectious agents that cause disease and characterizing population variations in these pathogens are essential for epidemiological surveillance and control decisions [13]. Previous molecular characterizations of PaWB phytoplasma concerning the sequence analysis and gene expression in *E. coli* of *tmk, ipt, tuf and* two plasmids; their function determinations; and *fusA-tuf* intergenic region analysis have provided useful molecular markers for genetic diversity and evolution analysis of phytoplasmas [14,15,16,17]. Cao et al. [7] reported the genome sequence of PaWB phytoplasma, finding that the effector PaWB-SAP54 mediates PaWB disease formation. However, the difficulty of culturing phytoplasma in vitro is an obstacle to elucidating the genetic variation and phylogenetic relationship among Chinese different geographical populations of PaWB disease. At present, due to the lack of efficient methods and inadequate collection of pathogenic strains, basic and applied studies on the pathogenicity, pathosystem, spread, disease management, and control decisions have been considerably hindered.

Multilocus sequence typing was first applied to the study of natural variation strains of *Neisseria meningitidis* [13], and then later applied to other pathogenic fungi, bacteria, and environmental microorganisms. MLST has become a classic method for epidemiological investigation, identification, transmission traceability, and genetic evolution analysis of pathogenic microorganisms [18]. Many MLST studies have also been conducted on the differential diagnosis of phytoplasmas and genetic diversity [19,20,21,22,23,24,25], such as for Flavescence dorée (FD) phytoplasma [26,27,28]. Jernej et al. [29] analyzed the occurrence of ‘*Candidatus Phytoplasma pyri*’ in different orchards in Slovenia, and the results showed that 45 samples from different sources could be divided into eight STs by MLST analysis of *secY* and *aceF*. These eight STs could be distinguished into two evolutionary branches, producing a more accurate distinction than 16S/23 S, but these STs did not show any correlation with host varieties. Abeysinghe et al. [30] verified that a new set of universal phytoplasma primers amplify approximately 1 kb of the leucyl transfer RNA synthetase (*leuS*) gene, which was used along with partial sequences of the *secA* gene to clarify the taxonomic classification of 16SrXI and 16SrXIV phytoplasmas and found that the sugarcane white leaf and grassy shoot phytoplasmas appeared to be the same phytoplasma. By means of multilocus sequence analyses using the 16S rRNA, 16S–23S intergeneric spacer region, *secA*, *secY*, and *groEL* of palm-infecting phytoplasmas in Florida, USA, Mou et al. confirmed the identity of lethal bronzing (LB) and lethal yellowing (LY) in the respective new hosts. Additionally, the novel molecular data (*secY*) helped to further understand and identify phytoplasmas in palms [31].

Yu et al. [32] firstly established the MLST technique for 16SrI phytoplasmas by analyzing the MLST of 16S rRNA and 10 housekeeping genes, including *rp*, *rpoB*, *gyrB*, *dnaK*, *tuf*, *secA*, *secY*, *ipt*, *pyrG*, and *fusA*, and revealed the diversity of phytoplasmas causing different plant diseases, such as mulberry dwarf, chinaberry witches’-broom, lettuce yellows, and periwinkle phyllody, as well as three PaWB isolates from the 16SrI group in different regions in China. They clearly divided the closely related phytoplasma, such as PaWB, CWB, MD, and LY, into different evolutionary lineages, then further divided CWB isolates from different regions into four evolutionary branches. In the study of Yu et al., the PaWB phytoplasma samples only originated from Shandong, Jiangsu, and Jiangxi provinces, so they failed to reveal the diversity of the overall population of PaWB that is widespread in China. Therefore, in this study, we used 16S rRNA, *rp*, *fusA*, *secY*, *tuf*, *secA*, *dnaK*, *rpoB*, *pyrG*, *gyrB*, and *ipt* genes to analyze the genetic structure of the population of PaWB phytoplasma strains collected from different geographical distributions by MLST to clarify the genetic diversity among different strains and analyze the phylogenetic relationship between PaWB phytoplasma and its closely related phytoplasma strains. This would give insights into the origin and pathways of the spread of PaWB phytoplasma.

## 2. Materials and Methods

### 2.1. Plant Material Collection and DNA Preparation

**Figure 1:**
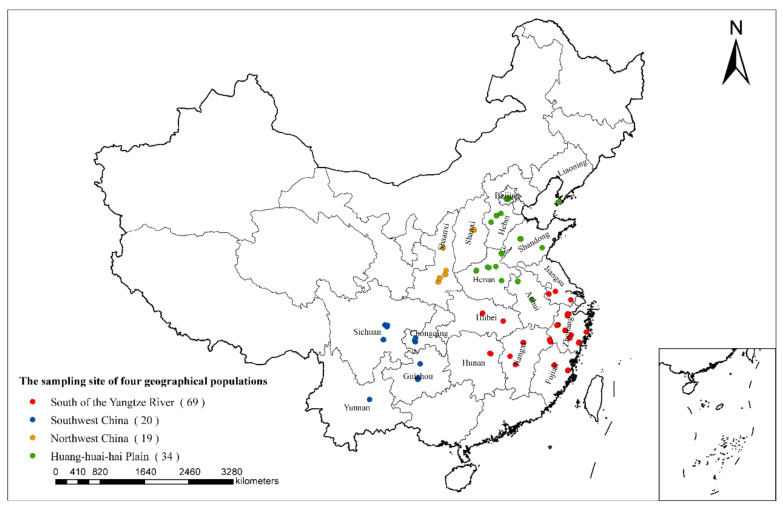
Geographical distribution of the 142 PaWB phytoplasma strains from four geographical populations, south of the Yangtze River population (69 strains), southwest China population (20 strains), northwest China population (19 strains), Huang-huai-hai Plain population (34 strains).

142 paulownia leaf samples with typical witches’-broom symptoms were collected from 18 provinces or municipalities in China (Table S1). The phytoplasma-free tissue culture seedlings of paulownia preserved in the laboratory were used as negative controls. The collected host plant paulownia species included *Paulownia fortunei*, *P. elongata*, *P. tomentosa*, *P. catalpifolia*, *P. taiwaniana*, *P. kawakamii*, *P. fargesii*, and unidentifiable hybrids. According to the cultivation conditions and climatic characteristics of paulownias in China, the PaWB phytoplasma samples were divided into four geographical populations: Huang-huai-hai Plain, northwest China, south of the Yangtze River, and southwest China (Figure 1). All the paulownia leaf samples were stored at −20 °C until required. Total DNA was extracted from the leaves of diseased paulownia by using a DNA extraction kit (CTAB Plant Genome DNA Rapid Extraction Kit, Aidlab Biotechnologies Co., Ltd., Beijing, China) [33]. The DNA samples were stored at −20 °C until use. DNA and related plant materials were stored at the Research Institute of Forest Ecology, Environment and Protection, Chinese Academy of Forestry.

### 2.2. Selection and Amplification of Housekeeping Genes

According to the conservation of different genes, some conservative single-copy genes as candidate genes were used for multigene sequence analysis. In this study, according to the results obtained by Yu et al. [32], we selected 16S rRNA and 10 housekeeping genes, including *rp* (encoding ribosomal protein), *fusA* (elongation factor G), *secY* (preprotein translocase subunit SecY), *tuf* (elongation factor Tu), *secA* (preprotein translocase subunit SecA), *dnaK* (molecular chaperone DnaK), *rpoB* (DNA-directed RNA polymerase subunit beta), *pyrG* (CTP synthase), *gyrB* (DNA gyrase subunit beta), and *ipt* (tRNA delta(2)-isopentenyl-pyrophosphate transferase). All primer sequences are shown in Table 1.

PCR amplifications of eleven genes were performed in 30 μL volumes containing 0.75 μL (10 μM) of each primer, 15 μL 2 × Taq PCR Master Mix (Aidlab Biotechnologies Co., Ltd., Beijing, China), 12.5 μL ddH_2_O and 1 μL undiluted DNA preparation. The reaction conditions were as follows: an initial denaturation step of 94 °C for 5 min, followed by 35 cycles consisting of denaturation at 94 °C for 30 s, annealing at 52 °C (for rp(I)FIA/rp(I)RIA and YugyrBf/YugyrBr), or 53 °C (for P1/P7, YufusAf/YufusAr, fTuf1/rTuf1, secAfor1/secArev3, and YupyrGf/YupyrGr), or 55 °C (for AYsecYF1/AYsecYR1, YudnaKf/YudnaKr, YurpoBf/YurpoBr, and iptf-BamHI/iptr-SalI) for 40 s; extension at 72 °C for 2 min (for P1/P7), or 1 min 20 s (for rp(I)FIA/rp(I)RIA, YudnaKf/YudnaKr, YurpoBf/YurpoBr, and YupyrGf/YupyrGr), or 1 min 30 s (for AYsecYF1/AYsecYR1, and YugyrBf/YugyrBr), or 1 min (for YufusAf/YufusAr, fTuf1/rTuf1, secAfor1/secArev3, and iptf-BamHI/iptr-SalI); with a final extension step of 72 °C for 10 min. The PCR products were visually detected by 1% (*w*/*v*) agarose gel electrophoresis through ethidium bromide staining. The nucleotide sequences of purified PCR products were determined by automated sequencing of both strands to achieve a minimum of 4-fold coverage per base position. DNA sequencing was carried out by a commercial DNA sequencing service (BGI Co., Ltd., Beijing, China).

### 2.3. PaWB Phytoplasma Diversity Analysis

The nucleotide sequences obtained in the study were assembled and edited using DNAMAN software, version 9.0 (Lynnon Corporation, Vaudreuil-Dorion, Quebec, QC, Canada). The multiple sequences of each gene were performed by DNAMAN 9.0 software to compare the genetic variation sites and similarities of different phytoplasma genes. The nucleotide sequences were compared and analyzed to identify the pathogen strains using the basic local alignment search tool (BLAST) (https://blast.ncbi.nlm.nih.gov/Blast.cgi (accessed on 30 July 2022)), depending on the sequence databases. The number of variable sites, the number of haplotypes, haplotype diversity, nucleotide diversity, and nonsynonymous-to-synonymous substitution ratio (*K_a_*/*K_s_*) were determined using DnaSP version 5.0. Tajima’s D [39], Fu and Li’s D, and Fu and Li’s F were performed on each housekeeping gene and the concatenated sequences with DnaSP software to determine whether the mutations in the sequence conformed to the neutral evolution theory of molecular evolution. The genetic differentiation index (*Fst*) and gene flow (*Nm*) of the different geographical populations were also analyzed using DnaSP software. Mismatch distribution was used to identify historical processes between recent demographic expansion and population equilibrium, which are characterized by smooth unimodal and multimodal distributions, respectively [40]. The mismatch analysis was implemented in DnaSP software.

### 2.4. Split Network and Recombination Analysis

The analyzed housekeeping genes underwent linkage disequilibrium analysis (the nonrandom association of alleles). The index of association (I_A_) is a measure of the degree of association between loci [41]. The standardized index of association (I_A_^S^) was calculated with Lian-Linkage Analysis, Version 3.7 (http://guanine.evolbio.mpg.de/cgi-bin/lian/lian.cgi.pl/) (accessed on 20 July 2022) [42]. The split networks were constructed by using SplitsTree4 v4.18 to evaluate the effect of recombination on phylogeny, and recombination was tested for each gene and for the concatenated sequences by performing the pairwise homoplasy index (*phi*) test [43,44]. Seven detection methods of RDP5 v5.23 (http://web.cbio.uct.ac.za/~darren/rdp.html) (accessed on 21 July 2022) were used to estimate the recombination of concatenated sequences, and at least four methods detected the significant recombination as valid [45].

### 2.5. Molecular Typing and Phylogeny of PaWB Phytoplasmas

We failed to amplify all the genes from the 142 strains. Thus, after screening, we analyzed the 105 strains’ concatenated sequences for seven housekeeping genes with good amplification effects. We assembled the concatenated sequences (*dnaK* + *fusA* + *rp* + *rpoB* + *secA* + *secY* + *tuf*) with SequenceMatrix1.7.8 [46]. Due to the lack of online shared MLST database for phytoplasmas, the STs of 105 PaWB phytoplasma strains were determined by referring to the MLST protocol of bacterial strains on the PubMLST website (https://pubmlst.org/organisms) (accessed on 8 June 2022). The allele number (haplotype number) of seven housekeeping genes in each strain was combined to form the allelic profile of the strain (Table S2), where each ST corresponded to the unique allelic profile. We considered strains with the same allelic profile to be of the same ST. Phylogenetic analysis of MLST data was performed with the goeBURST algorithm from PHYLOViZ 2.0 (http://www.phyloviz.net/) (accessed on 16 July 2022) [47,48]. The minimum spanning tree (MSTree) based on Prim’s algorithm was constructed using BioNumerics v7.6 software (Applied-Maths Corporation, Sint-Martens-Latem, Belgium) software to analyze the relationship between STs and source regions.

Phylogenetic analysis was performed with MEGA software, version 7.0 [49], employing the maximum-likelihood (ML) method with 1000 bootstrap values. The control strains were composed of six kinds of phytoplasmas: aster yellows witches’-broom (AYWB), onion yellows phytoplasma (OY-M), lettuce yellows phytoplasma (LY-fjsm1 and LY-fjsm2), periwinkle virescence phytoplasma (PeV-hnhk), chinaberry witches’-broom phytoplasma (CWB-fjya and CWB-jxnc), and mulberry dwarf phytoplasma (MD-ahhf and MD-zjca) (Table S3).

### 2.6. Nucleotide Sequence Accession Numbers

The DNA sequences identified in this study were all deposited in the GenBank database using the Portal (GenBank) tool for submission of the ribosomal RNA (rRNA) sequences (https://submit.ncbi.nlm.nih.gov/about/genbank/ (accessed on 30 July 2022)) and the Bankit tool for the submission of the protein-coding genes (https://submit.ncbi.nlm.nih.gov/about/bankit/ (accessed on 31 July 2022)). The GenBank accession numbers of the DNA sequences in the study are shown in Table S1.

## 3. Results

### 3.1. Genetic Diversity of 16S rRNA Gene and Different Housekeeping Genes in PaWB Phytoplasma

The results of the BLAST search, based on the 16S rRNA genes of the PaWB phytoplasmas, indicated that the 16S rRNA genes of the 122 PaWB phytoplasma strains had sequence identities, ranging from 99.76% to 100% (with sequences covering > 99.92%) when compared to the reference PaWB–Zhengzhou strain (GenBank accession number CP066882). Because not all 142 PaWB phytoplasma strains were amplified to obtain the corresponding sequences, the obtained number of sequences that we used for analyzing the genetic variation of each gene also somewhat differed (Table 2).

From the available sequences in Table 2, the amplification efficiencies of *rp*, *fusA*, *secY*, and *tuf* genes were relatively high, and the number of complete sequences was more than 133, accounting for more than 93.66% of all strains. The amplification efficiencies for *gyrB* and *ipt* genes were slightly lower, and the number of complete sequences did not exceed 100. The GC content of the 10 housekeeping genes ranged from 30.3% to 37.7%, which was similar to that of the reference PaWB–Zhengzhou strain (CP066882), indicating that the 10 genes were highly conserved over the long-term evolution of PaWB phytoplasma. The sequence similarity of each housekeeping gene was above 99%, among all the sequenced strains.

Comparing the numbers of single-nucleotide polymorphisms (SNPs) of the 16S rRNA gene and 10 housekeeping genes with DnaSP software (Tables S4–S14), the number of gene variation sites for the different housekeeping genes ranged from 1 to 14 SNPs. The *rp* gene of six strains had a single codon (threonine) deletion (Table S5). The strains were collected from Shaanxi province (4 strains) and Jiangxi province (two strains). The genetic variation of *rpoB* (1.47%) and *dnaK* (1.12%) was higher, and that of the *rp* (0.20%) gene was the lowest. The number of haplotypes ranged from 2 (*rp*) to 17 (16S rRNA). Among them, six genes (*fusA*, *secY*, *tuf*, *dnaK*, *pyrG*, and *gyrB*) had strong identification ability, which could classify most strains from Fujian and Jiangxi and individual strains from Shaanxi into the same haplotype. The haplotype diversity of the ribosomal gene (16S rRNA) was substantially higher than that of each functional housekeeping gene. The ribosomal gene sequences showed higher genetic diversity in PaWB phytoplasma strains from different regions in China.

The *K_a_*/*K_s_* ratio (the average nonsynonymous rate (*K_a_*) to the average synonymous rate (*K_s_*)) ranged from 0 (*rp*) to 2.63 (*tuf*), except for the *gyrB* gene. The *K_a_*/*K_s_* ratios of *tuf* and *rpoB* genes were considerably larger than one, so it was judged that the two genes might have mutated under positive selection pressure. The mutations in the other genes were the result of negative or purification selection. The results of the neutral evolution test of different genes showed that the detection of most genes was not remarkable, and their evolution was in line with neutral evolution theory. Only Fu and Li’s D detection and Fu and Li’s F detection results of *tuf* were significant (*p* < 0.05) and their values were significantly negative, so we inferred that *tuf* might have been subjected to directional selection.

### 3.2. Geographical Distribution and Population Genetics Analysis of PaWB Phytoplasma

#### 3.2.1. Genetic Diversity of PaWB Phytoplasma in Different Geographical Populations

We detected 22 haplotypes and 42 variable sites in 105 concatenated sequences of PaWB phytoplasma with DnaSP software. The results of genetic diversity analysis showed that the total population diversity of STs was 0.474 and nucleotide diversity was 0.0009 (Table 3). South of the Yangtze River population had the most polymorphic sites. There was only one ST without any polymorphic sites in the southwest China population. The populations in the south of the Yangtze River and in the Huang-huai-hai Plain had the largest numbers of sequence types. Northwest China population had the highest STs diversity (0.724); south of the Yangtze River population had the highest nucleotide diversity (0.00153). Furthermore, Tajima’s D and Fu and Li’s D and F of the concatenated sequences were calculated for each geographical population and at the national level in China. Tajima’s D and Fu and Li’s D and F for northwest China and Huang-huai-hai Plain populations were negative and statistically significant, indicating that these two populations were affected by directional selection.

The STs distribution illustrates their aggregation in each geographical population. Among them, ST1 appeared in all four geographical populations, having the widest distribution and accounting for 72.38% of all samples. ST1 had a high frequency of shared sequence type and was the dominant sequence type of PaWB phytoplasma in China.

#### 3.2.2. Population Genetics and Mismatch Distribution Analysis of PaWB Phytoplasma

Based on the multilocus sequence data of the four geographical populations in China, the population genetic differentiation index (*Fst*) and gene flow (*Nm*) among the populations of PaWB phytoplasma in China were analyzed with DnaSP software (Table 4 and Table 5) [50]. The results showed that the *Fst* ranged from 0 to 0.23148, and the *Nm* ranged from 0 to 37.53. The *Fst* between the populations in south of the Yangtze River population and in southwest China was the largest (0.23148). The degree of genetic differentiation between the populations in the south of the Yangtze River and in the Huang-huai-hai Plain (or Southwest China) was relatively large. The gene flow may have occurred among the four populations, reaching up to 1.66, even between the populations in the south of the Yangtze River and in southwest China. According to Wright’s Shifting Balance theory, the gene flow between populations is larger than one; the genetic differentiation caused by genetic drift is effectively inhibited. When the *Nm* value is larger than four, the gene exchange between populations is frequent and sufficient [51]. Therefore, it could be determined that the gene flow was strong between the PaWB phytoplasma strains of the different geographical populations in China.

**Figure 2:**
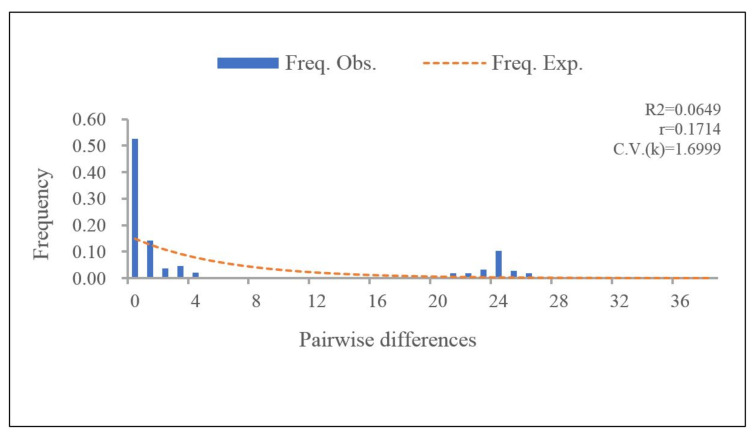
Pairwise mismatch distributions for 105 PaWB phytoplasma strains, based on the concatenated sequences by DnaSP software. The *X*-axis shows the observed distribution of pairwise genetic variation, and the *Y*-axis represents the frequencies.

For the PaWB phytoplasma population in China, there were no explicit signals of population expansion or equilibrium, as the results of the neutral test of concatenated sequences was not significant and the observed mismatch distribution identified multimodal (Figure 2). We inferred that the growth of the PaWB phytoplasma populations was not sustained: the populations size was relatively stable in recent years.

### 3.3. Allelic Sequences Recombination Analysis

The Lian-Linkage3.7 software [42] was used to analyze the recombination of seven housekeeping genes. If clonal structures are preset between alleles, I_A_^S^ is significantly different from zero. If the alleles are randomly associated, I_A_^S^ is closer to zero. The results showed that the I_A_^S^ value of the seven housekeeping genes was 0.6091 (*p* < 0.01), indicating clonal structures and rare recombination between alleles, which is linkage disequilibrium.

**Figure 3:**
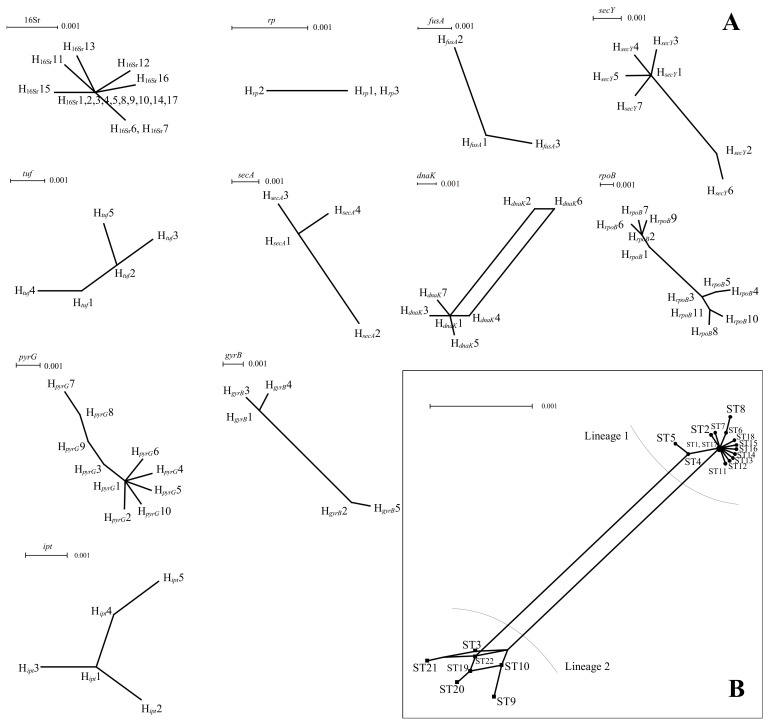
(**A**) Split network of PaWB phytoplasma strains based on each housekeeping gene. (**B**) Split network of PaWB phytoplasma strains based on concatenate sequences of 7 housekeeping genes.

To further explore the effect of recombination on the population structure of PaWB phytoplasma, split networks were calculated from the PaWB phytoplasma sequences for the 16Sr RNA genes and 10 housekeeping genes and for the concatenated sequences (Figure 3). Only *dnaK* showed a parallelogram structure, and the other 10 genes (16Sr RNA, *rp*, *fusA*, *secY*, *tuf*, *secA*, *rpoB*, *pyrG*, *gyrB*, and *ipt*) showed a dendritic structure, indicating that these genes had not or rarely recombined during evolution. The results of the *phi*-test analysis of seven housekeeping genes showed that only *dnaK* had significant recombination (*p* = 0.042; Table 3). No recombination was found among the other 10 housekeeping genes. The results of the analysis of the concatenated sequences showed that 22 STs had two genetic lineages (i.e., lineage-1 and lineage-2) and showed a partial network structure (Figure 3), but the results obtained using RDP v5.23 did not identify any recombination signals.

### 3.4. Evolutionary Analysis with goeBURST Algorithm

**Figure 4:**
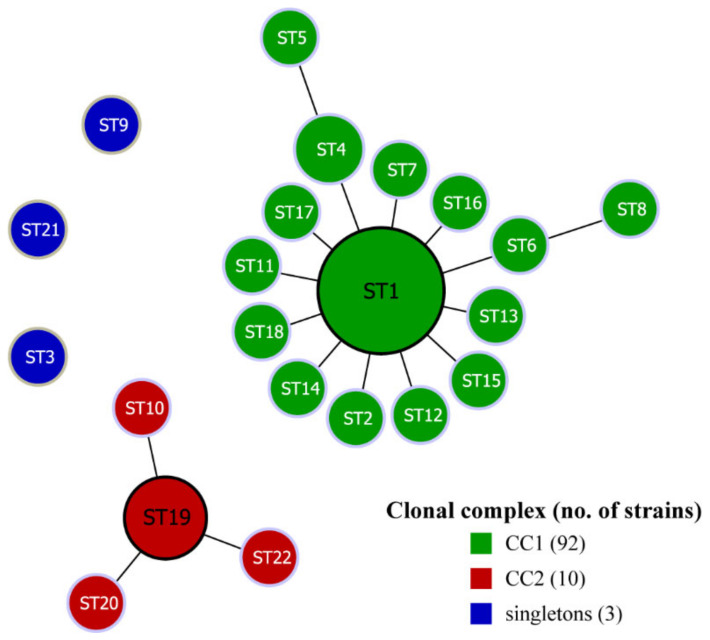
The goeBURST analysis of 105 PaWB phytoplasma strains. Defined at SLV level to the clonal complexes (groups). Each ST is represented by a circle; the size of the circle is proportional to the number of strains.

Assessing the PaWB phytoplasma population organization and the patterns of evolution, the linked STs that shared 6/7 alleles (single locus variants (SLVs)) were clustered together by the goeBURST algorithm into genetically related groups referred to as a clonal complex (CC). The 22 STs of the 105 PaWB phytoplasma strains were analyzed with the goeBURST algorithm and formed two clonal complexes and three singletons (Figure 4). Among them, CC1 was the most notable CC, which was composed of ST1, 12 SLVs, and 2 double-locus variants (DLVs). CC1 had the largest number of strains, including 92 PaWB phytoplasma strains in 15 STs, accounting for 87.6% of all strains. In addition, the ST1 in the clonal complex was the most frequent sequence type, including the largest number of strains and located at the center of CC1. Therefore, ST1 was considered as the primary founder, and the other STs in CC1 might have evolved with ST1 as the center. CC2 was composed of ST10, ST19, ST20, and ST21, including 10 strains, and ST19 was located in the center, which was the primary founder of CC2.

### 3.5. Minimum Spanning Tree Analysis

**Figure 5:**
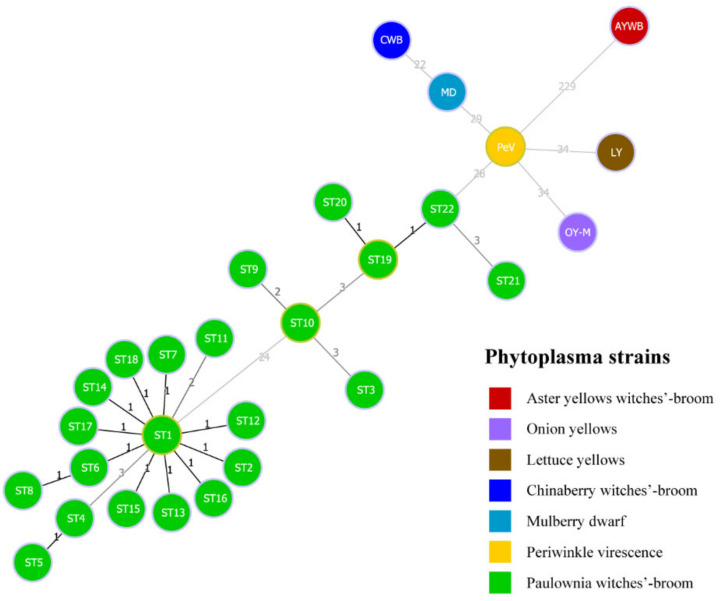
The population snapshot of PaWB phytoplasma strains and 6 kinds of phytoplasma reference strains, diagrammed based on a minimum spanning tree using goeBURST algorithm of PHYLOViZ 2.0 software, based on the concatenated sequences. Green circles represent 22 STs of 105 PaWB phytoplasma strains. The numbers above links represented the numbers of single-nucleotide polymorphisms (SNPs) between STs (including reference strains).

A minimum spanning tree was constructed with the goeBURST algorithm of PHYLOViZ 2.0 software to analyze genetic evolution between the STs of PaWB phytoplasma strains and six kinds of phytoplasma reference strains in the 16SrI group (Figure 5). From the tree, PaWB phytoplasma strains were distinguished from six kinds of reference strains at the SNPs level. From the tree, MLST-concatenated sequences of the samples of PaWB phytoplasma were divided into two groups, and the difference between them was 24 SNPs. ST1 and ST19 were the central nodes of the two separate PaWB phytoplasma groups.

**Figure 6:**
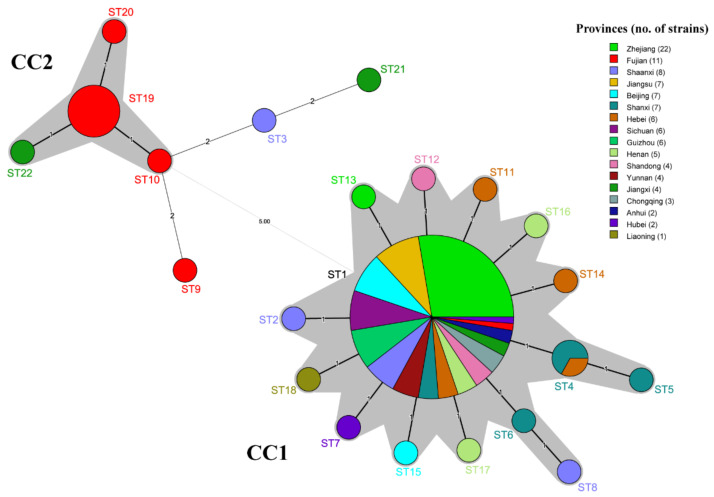
Minimum spanning tree of STs of 105 PaWB phytoplasma strains based on different provinces. Each circle represents a sequence type (ST). The shadow part was the two clonal complexes. The black thick solid lines indicated the closest genetic relationship, the black thin solid lines indicated that the closer genetic relationship, and the dotted lines indicated a further genetic relationship. The number above links represented the number of locus differences, and the size of circles represented the number of strains.

To further analyze the population structure of PaWB phytoplasma strains, the minimum spanning tree was constructed with Prim’s algorithm of BioNumerics v7.6 software (Figure 6). The provinces of the PaWB phytoplasmas are represented by different colors for each ST node. The structure of the MSTree illustrates both regional differentiation and the clonal structure of the PaWB phytoplasma populations. The 22 STs formed two branches, and the left branch contained 13 strains from CC2 and 3 singletons. The strains were collected from Fujian (10 strains), Jiangxi (2 strains), and Shaanxi (1 strain); the 92 strains of the right branch were all from the CC1, with ST1 as the primary founder of CC1, being distributed in all sampled provinces (except Liaoning). The strains from the southwest China population were collected from Chongqing, Sichuan, Guizhou, and Yunnan provinces, all belonging to the ST1. Comparing the results in Figure 6 with the MSTree in Figure 5, ST21 is closer to ST3 than to ST22 in evolution because there are two loci differences (including five SNPs) between ST21 and ST3, and three loci differences (including three SNPs) between ST21 and ST22.

### 3.6. Phylogenetic Analysis of 16S rRNA Gene Sequences and Concatenated Sequences

**Figure 7:**
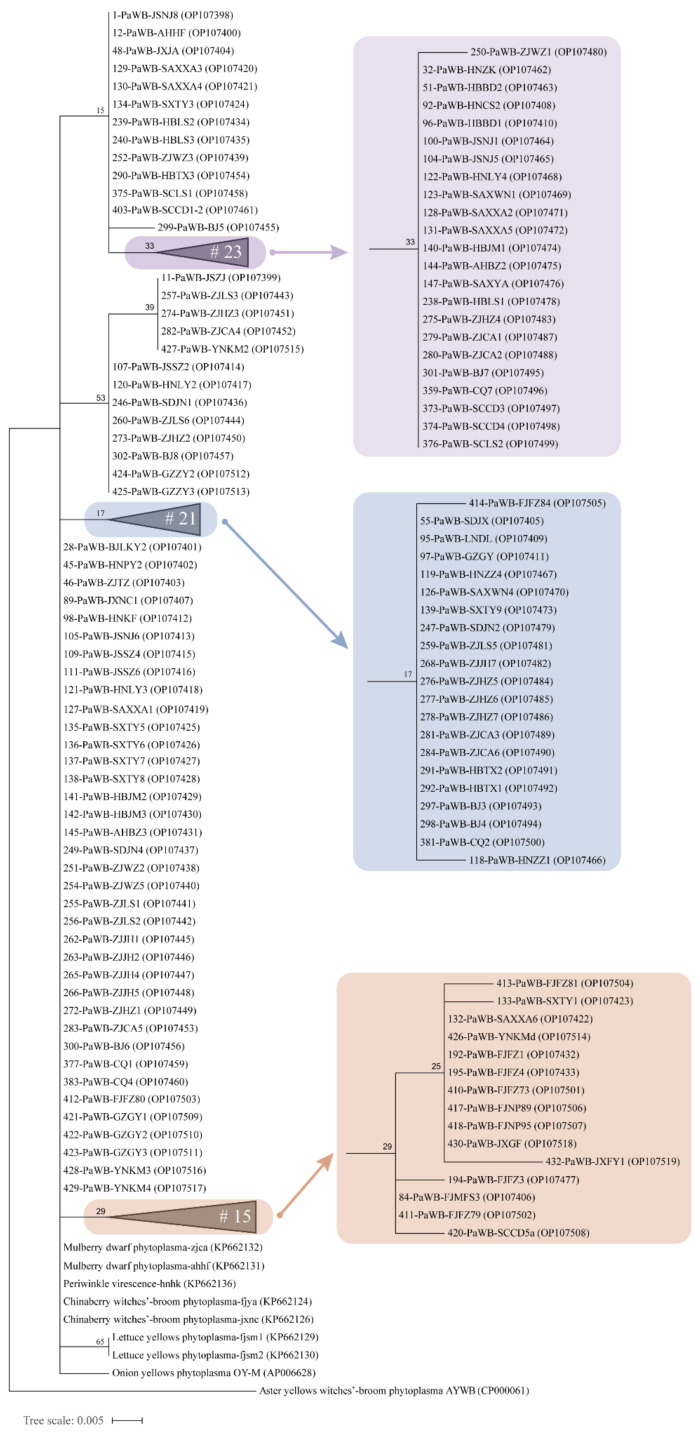
Maximum-likelihood phylogenetic tree for the 105 PaWB phytoplasma strains and 6 kinds of phytoplasma reference strains based on the 16S rRNA gene sequences. The collapsed clades are displayed as triangles, and the total number (#) of strains is shown within the triangles. The scale bar length represents inferred character-state changes under the best-fitting JC + G model. Branch lengths are proportional to the number of inferred character-state transformations. The percentage of replicate trees in which the associated taxa clustered together in the bootstrap test (1000 replicates) is shown next to the branches.

The phylogenetic tree based on the 16S rRNA gene sequences showed that the phytoplasma strains from different hosts, such as PaWB, chinaberry witches’ broom (CWB), mulberry dwarf (MD), and periwinkle phyllody (PeV), clustered on a branch (Figure 7). OY-M and lettuce yellows (LY) were located in independent branches, where the branch of two LY phytoplasma strains had 65% bootstrap support. Thirteen PaWB phytoplasma strains from Jiangsu, Zhejiang, Henan, Beijing, Shandong, Yunnan, and Guizhou provinces were clustered together with 53% bootstrap support. In addition, 9 of the 11 strains collected from Fujian clustered in the same clade (the marked part in light brown in Figure 7). There was no or low bootstrap support for analyzing genetic diversity and regionality by the 16S rRNA gene sequences among the strains sampled from most regions.

**Figure 8:**
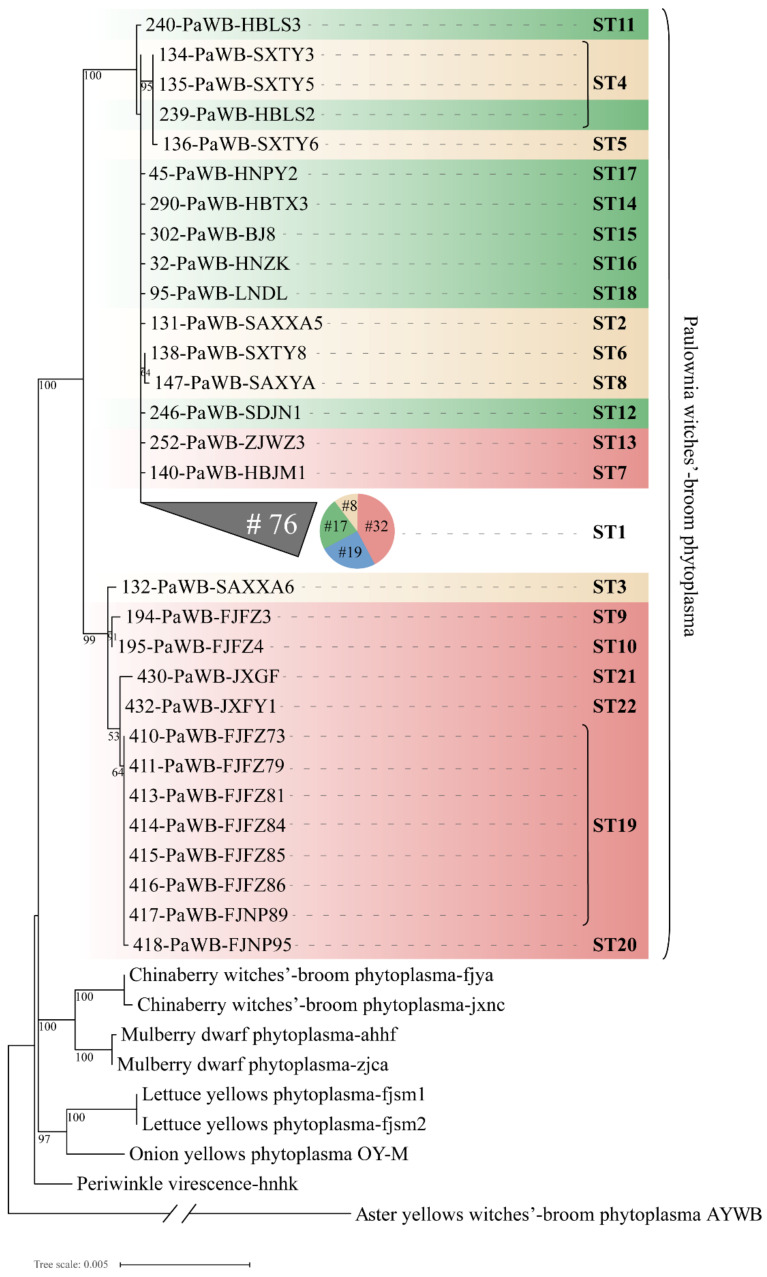
Phylogenetic tree constructed based on the concatenated sequences of the 105 PaWB phytoplasma strains using 7 housekeeping genes and 6 kinds of phytoplasma reference strains employing the maximum likelihood method. Four colors represent the geographical populations of PaWB phytoplasmas: Huang-huai-hai Plain population is green color gradient, northwest China population is yellow color gradient, south of the Yangtze River population is red color gradient, southwest China population is blue. The collapsed clade is displayed as a triangle, and the number (#) of strains included in the collapsed clade is indicated. The scale bar length represents inferred character-state changes under the best-fitting T92 + G model. Branch lengths are proportional to the number of inferred character-state transformations. The percentage of replicate trees in which the associated taxa clustered together in the bootstrap test (1000 replicates) is shown next to the branches. An expanded phylogeny is provided in Figure S1.

The results of the phylogenetic analysis of the concatenated sequences of seven housekeeping genes employing the maximum likelihood method showed that the phytoplasma strains of different hosts could be divided into different branches with higher bootstrap support (Figure 8). The 105 PaWB phytoplasma strains from different regions were geographically differentiated and were divided into two main clades. Among them, ten, two, and one strains collected from Fujian, Jiangxi, and Shaanxi provinces, respectively, were clustered in the same clade with 99% bootstrap support. These strains, except for one from Shaanxi in the clade were from the south of the Yangtze River geographical population (the red color gradient in Figure 8). The PaWB phytoplasma strains from the southwest China population were distributed in the collapsed clade. The phylogenetic tree presents a topology similar to that of the minimal spanning tree using the goeBURST (Figure 5), which also explains the remarkable genetic differentiation between the paulownia phytoplasma strains collected from Fujian, Jiangxi, and Shaanxi provinces and the strains from other regions. The maximum likelihood phylogenetic tree for the 22 STs of PaWB phytoplasmas based on the concatenated sequences of six proteins (except ribosomal protein) (Figure S2) showed that the MLST results of concatenated sequences of six proteins were in agreement with those of the nucleotide sequences, despite some differences in the topological branching structure.

### 3.7. Phylogenetic Analysis of Each Housekeeping Gene in PaWB Phytoplasma

Phylogenetic trees were constructed based on the 10 housekeeping genes with the maximum likelihood method (Figures S3–S12). In addition to the phylogenetic tree constructed by the *tuf* gene sequences, the phylogenetic trees constructed by the other nine housekeeping genes could more accurately distinguish the phytoplasma strains between the different hosts and with strong bootstrap support. In the phylogenetic trees based on each housekeeping gene, except for *rp*, *rpoB*, *secA*, and *tuf*, the similarity between the LY phytoplasma in Fujian and the OY-M phytoplasma in Japan was high, which could be clustered into a branch. For all the phylogenetic trees of the housekeeping genes, the PaWB phytoplasma strains from the different regions in China were difficult to distinguish geographically; the phylogenetic tree constructed by the *rp* gene sequences, in particular, showed that almost all PaWB phytoplasma strains were clustered into a complete branch. The other housekeeping genes with discrimination ability produced a common result: there were differences between most PaWB phytoplasma strains from Fujian and Jiangxi provinces and the strains from most parts of the regions in China and their similarity with individual strains from the Shaanxi province was high, which clustered into a clade with strong bootstrap support.

The phylogenetic tree constructed based on 10 housekeeping genes was compared with that of the 16S rRNA gene. The evolutionary analysis with housekeeping genes as molecular markers better differentiated the phytoplasma strains among the different host plants. Due to the relative conservativeness of each housekeeping gene in the same phytoplasma, we found fewer genetic variation sites than the differences in the phytoplasma strains from different regions and with climatic conditions to a certain extent.

## 4. Discussion

### 4.1. PaWB Phytoplasma as a Unique Phylogenetic Branch of Group 16SrI That Is Closely Related to 16SrI-B

Based on the sequence data of the 16S rRNA and 10 housekeeping genes, all PaWB phytoplasma strains were identified as taxonomic subgroup D within the 16SrI group, ‘*Ca*. P. asteris’-member strain clusters, in accordance with the results of previous reports in China, Japan, and Korea [9,10,32]. The evidence supported that PaWB phytoplasma strains can be assigned to a unique phylogenic clade in 16SrI, which is most similar to the members of the subgroup16SrI-B phytoplasmas occurring in China, which includes mulberry dwarf, chinaberry witches’-broom, periwinkle virescence, and lettuce yellows phytoplasmas, as well as onion yellows phytoplasma in Japan, but are genetically distant from AYWB phytoplasma from North America, which is assigned as the 16SrI-A subgroup [9]. Therefore, it is assumed that both phytoplasma strains of the 16SrI-B members and PaWB 16SrI-D strains occurring in Asian regions more likely shared an identical ancestor than 16SrI-A strains represented by the ‘*Ca. P. asteris*’ species during bacterium evolution history, owing to the various gene flows, adaptation, or geographical isolation. So far, no mixture infection or co-occurrence of PaWB 16SrI-D phytoplasma with other subgroup strains of 16SrI or any other group have been evidenced, as reported for other diseases [52,53,54]. Although Wang et al. detected PaWB phytoplasma from samples of several plants with atypical symptoms around a plantation with PaWB disease; however, their role in the occurrence remains unknown [15]. Thus, *Paulownia* spp. are thought to be an important plant host of PaWB phytoplasma, causing problems with this disease.

### 4.2. Valuable Methods for PaWB Phytoplasma Traceability and Disease Epidemics Based on MLST Scheme

As phytoplasma strains cannot be cultured in vitro, studying the genetic diversity of phytoplasma strains based on whole-genome SNP technology is difficult, and discriminating phytoplasmas from different hosts and regions only by RFLP analysis based on the 16S rRNA sequences, which have insufficient mutation sites and low resolution, is challenging. The newly emerged MLST technology can be used to overcome the shortcomings of RFLP analysis and improve the resolution between phytoplasma strains [55,56,57]. On the basis of the MLST approach, the combination of goeBURST algorithm analysis, minimum spanning tree analysis, and ML phylogenetic tree analysis not only enables the finer identification and differentiation of PaWB phytoplasma strains under the levels of subgroup or *Candidatus* species but also has the potential to distinguish geographic variability, which will be helpful for long-term or short-term epidemiology.

In this study, the results of the 16S rRNA gene sequences analysis of PaWB phytoplasma showed that the sequences were relatively conservative; although they showed certain variability, they did not easily reflect the evolutionary relationship in more detail among different strains when strains were indistinguishable, owing to their close genetic relationship. Based on the MLST scheme, we found two representative lineages of PaWB phytoplasma. We consider the dominant sequence, ST1, of the PaWB phytoplasma as the primary founder, representing the highly homogeneous lineage-1 (CC1). This may reflect that the PaWB epidemics have been mainly caused by the strain cluster of CC1, which has been widely distributed in most disease-affected areas. However, we found lineage-2 (including CC2 and three singletons) of PaWB phytoplasma in the Fujian and Jiangxi provinces, where the ST19 strains of CC2 were the predominant strains in the Fujian province. The explanation for the large-scale distribution of diseases associated with ST1 strains should involve consideration of some factors, including the widespread vegetative (clonal) propagation used in paulownia cultivation, such as root-cutting and seedlings, the planting of susceptible species or varieties, and the environmental conditions suitable for disease spread and development [3,58]. When collecting samples in the south of the Yangtze River, we also found that the symptoms of *P. fortunei* and *P. taiwaniana* infected with the CC2 strains were different from the usual PaWB symptoms. Therefore, further study of the variation, origin, and virulence of this strain lineage is necessary, and the relationship between this strain cluster and other phytoplasma strains in most regions needs to be revealed. In addition, because we failed to find *Halyomorpha halys* (Stål) distributed in the locations in southern China during the survey in recent years, which is the main insect vector of PaWB disease in the Huang-huai-hai Plain and the northwest regions (unpublished data), whether other different kinds of insect vector species, in addition to stinkbugs, are responsible for the spread of these variable strains should be examined.

We expect that, based on the MLST scheme, certain problems involving the PaWB pathosystem, epidemics, and management will be gradually solved, underlining the importance of the collection of more representative samples from the hot spots of genetic variability, of resistant and tolerant host species or varieties, symptomless samples, and the vector insects or other alternative host plants within disease stands or nearby. Furthermore, it is suggested that genotyping old historical phytoplasma samples is the most suitable method of redrawing the routes of introduction and evolution of the phytoplasmas [53].

### 4.3. Variation in PaWB Phytoplasma Housekeeping Genes Related to Geographical and Environmental Factors

Researchers previously found that the Huang-huai-hai Plain and northwest China were the areas most severely affected by PaWB disease, followed by southwest China and south of the Yangtze River [3]. The disease index in different regions negatively correlated with the mean annual temperature and average annual precipitation. Due to the fact that the total number of paulownia trees in southwest China is lower than in other regions and the relatively low incidence of PaWB disease, we only collected 20 samples of infected paulownia from southwest China, matching the disease situation there. The number of infected paulownia samples that were gathered in this region was also smaller because we only sampled from four cities representing the main paulownia cultivation and disease distribution in northwest China. As phytoplasma possesses dynamic genomes and rapidly evolves toward the formation of distinct ecological lineages in their adaptation to specific ecological niches [59], the high variation in the strains in Fujian and Jiangxi provinces, compared with that of the majority of strains causing regional or nationwide epidemics of the disease, may be due to long-term geographical isolation, regionality, and the resistance of host species or clones, as well as specified insect vector or environmental changes, resulting in suitability variation. For instance, the occurrence of PaWB disease in the Fujian province was most recently found in 2015, which is separated by Wuyi Mountain from the Yangtze River area and is closer to Taiwan Island. There had been a report about PaWB disease outbreaks in Taiwan province before the 1980s [60]. This raises an intriguing question as to whether the Fujian strains, possessing the largest genetic variation among all the strains on the mainland, share an identical origin as those in Taiwan. Additionally, the rather susceptible host species, *Paulownia taiwaniana*, is commonly distributed in both areas. The high homology of individual PaWB phytoplasma strains collected from Shaanxi with Fujian and Jiangxi strains may be related to phytoplasma immigration along with the introduction and breeding of large-scale paulownia germplasm resources in Shaanxi province after the 1980s [61]. Due to the immaturity of the PCR detection technology for pathogens at that time, asymptomatic paulownia seedlings carrying the phytoplasmas of CC2 may have been introduced into and then colonized in northwest China. The relatively effective treatments for PaWB disease include early eradication or breeding-resistant paulownia species. Paulownia species and varieties are diverse in China, and the regions in which one to several specialized species are being cultivated are distributed in different climates and geographical conditions. Although no species or variety of *paulowina* spp. is immune to PaWB disease and most are easily infected and show typical witches’-broom symptoms, some resistant and tolerant germplasms have been identified [3,58].

The reasons for the observed geographic patterns remain to be elucidated, but, to a certain extent, environmental stress conditions, such as drought, could have led to the host paulownia and pathogen forming a special interaction, increasing fitness in special habitats at the sieve tube site and promoting the diversification of phytoplasma strains in the northwest region. In addition, the gene exchange among strains during the introduction and breeding of large-scale paulownia germplasm resources in the Shaanxi province after the 1980s is also a factor that cannot be ignored. According to the Tajima’s D and Fu and Li’s D and F detection results of the concatenated sequences using the MLST scheme between the Huang-huai-hai Plain and northwest China populations, the gene variations of these two populations were subjected to directional selection, and our preliminary inference is that this was caused by breeding resistant tree species, selecting regional vector insects, or the impact of climate factors. Pilet et al. [53] reported that the occurrence of strong bottlenecks in the ‘*Ca. Phytoplasma palmicola*’ population in African countries might explain the low diversity at national or regional scales because the phytoplasma led to the rapid and inescapable death of the infected coconut in about 1 year and the new genetic phytoplasma variant had a short span of time to appear and emerge from the initial plant host. However, our findings showed that no obvious bottlenecks have recently occurred in the PaWB population in China: most infected paulownia trees seldom die rapidly and can survive for a long time.

Obviously, the number of samples we obtained for all 11 gene sequences is less than the total number of samples collected. The PCR amplification of all gene sequences of each sample was performed three times. However, in some infected paulownia leaf samples with unobvious witches’-broom symptoms collected in the field, the pathogen concentration of the samples was low, so the PCR of these samples had failed, or the PCR products of these samples showed overlapping peaks due to non-specific amplification in the sequencing results. In addition, very few samples had run out of DNA, and we could not finally get the sequence of all the genes in these samples. More gene sequences could be obtained from this portion of the samples if PCR optimization is carried out further. In this study, the results of the phylogeny analysis of nine functional housekeeping genes, with the exception of the *tuf* gene, allowed us to distinctly divide the 16SrI phytoplasma strains among the chosen seven different host plants, including paulownia. We think that this may be owing to *pyrG*, *ipt*, *secY*, and other genes being involved in the regulation of phytoplasma gene expression. In different host plants, owing to changes in living environment of the host, the host may interact differently with the pathogen, resulting in variation. In this study, we found that the strains of the dominant sequence, ST1, in China were the most widely distributed, and their hosts were mostly paulownia species. The host plants of the strains of lineage-2 were mainly *P. fortunei* and *P. taiwaniana*. These variations, indicating ecological niches and selection pressure or neutral mutation, may reveal the co-evolution relationship between the phytoplasma and host (plant and insect vector), as well as environment conditions.

The results of a study of PaWB *ipt* gene expression in *E. coli* and its function suggested that this gene may be involved in the disturbance of the cytokinin-like hormone of infected plants and in symptom development [38], which raises the question as to whether the variations in the *ipt* gene among the tested PaWB phytoplasma strains from the south of the Yangtze River population may have been affected by environmental factors. However, the PaWB *tuf* gene encodes the elongation factor, EF-Tu and is one of the most abundant and essential proteins in protein synthesis. The *tuf* gene has low genetic diversity, but its *K_a_*/*K_s_* ratio value is considerably larger than one, and its Fu and Li’s D and F values are negative (*p* < 0.05). It is inferred that the nucleotide mutation of *tuf* gene could have been mainly affected by natural selection, such as phytoplasma antigenic membrane protein genes (*amp*) that could determine insect–vector specificity through the interaction between the membrane proteins of the phytoplasma and the insect microfilament complex [62,63]. The antibody prepared through the expression of the PaWB *tuf* gene in the *E. coli* and LAMP primers set is not the only one that can be used for detecting PaWB phytoplasma strains; the members of 16SrI-B, such as chinaberry witches’ broom, mulberry dwarf, periwinkle virescence, and lettuce yellows phytoplasmas can also be used [15,64].

Pilet et al. [53], considering eight housekeeping genes, found no evidence of recombination events for ‘*Ca. Phytoplasma palmicola*’ related to geographical isolation. However, in this study, we found that the *dnaK* gene, which encodes the heat shock protein, showed sporadic recombination ability to help with adaptation to extreme temperature environments. The evidence of intermolecular recombination between the extrachromosomal DNAs of wildtype onion yellow phytoplasma wild type and mild-symptom lines further supports the idea that recombination has played a major evolutionary role by creating genetic diversity and indicates the potential for rapid adaptation to new environmental conditions [65]. The nonrecombinant of other genes may have been due to the strong clonality (i.e., linkage disequilibrium) of the housekeeping genes during the asexual reproduction of the strains, or their uneven distribution in the host, reducing the probability of recombination. At present, no interspecies co-infection has been detected, which requires further sampling and detection in areas where PaWB phytoplasma strains of CC1 and CC2 share the same geographical distribution, such as in northern Fujian and Jiangxi. As a conserved gene of ribosome synthesis, the 16S rRNA gene interacted less with different hosts, and its variation appeared to rarely reflect the interaction process between pathogen and host. Therefore, more variable functional housekeeping genes may be superior to ribosomal genes in distinguishing the phytoplasmas of different host plants.

## 5. Conclusions

In this study, we effectively determined that PaWB phytoplasmas and other subgroups of phytoplasma reference strains in 16SrI have substantial indigenous characteristics. We further confirmed the potential of the use of the MLST scheme for the fine identification and discrimination of pathogenetic bacterial strains within populations of 16S rDNA that are closely related and indistinguishable. The results of molecular diversity analysis of PaWB phytoplasmas from different geographical populations in China with MLST showed that the mutations in the *tuf* and *rpoB* genes were driven by some positive selection pressure, and the *tuf* gene did not follow the law of neutral evolution. The absence of recombination among the PaWB phytoplasma sequence types for each housekeeping gene, except for *dnaK*, might have been due to strong clonality. We divided the 105 PaWB phytoplasma strains into 22 sequence types with MLST, which had comparatively higher genetic diversity and were clustered into two representative lineages with bootstrap values larger than 99%. The CC1 strains were widely distributed in four geographical populations, while CC2 strains were mainly distributed in the south of the Yangtze River, with notable geographical differentiation. Using the representative MLST scheme to reveal the genetic variation in PaWB phytoplasma provided results that are beneficial for PaWB epidemiological monitoring and disease prevention through the fine differentiation and analysis of closely related phytoplasma strain lineages.

## Figures and Tables

**Table 1:** Universal and specific primers used to amplify each PaWB phytoplasma MLST locus.

Primer Pairs		Primer Sequences (5′-3′)	Amplicon Length (bp)	References
16S rRNA	P1	AAGAATTTGATCCTGGCTCAGGATT	1437–1440	Dickinson et al., 2013 [34]
	P7	CGTCCTTCATCGGCTCTT		
*rp*	rp(I)FIA	TTTTCCCCTACACGTACTTA	1080–1093	Lee et al., 2003 [35]
	rp(I)RIA	GTTCTTTTTGGCATTAACAT		
*fusA*	YufusAf	GTTGTTGACTACCTTCCTGCTC	914–926	Yu et al., 2017 [32]
	YufusAr	TCGCCAATAACATTTCCTAC		
*secY*	AYsecYF1	CAGCCATTTTAGCAGTTGGTGG	1215–1258	Dickinson et al., 2013 [34]
	AYsecYR1	CAGAAGCTTGAGTGCCTTTACC		
*tuf*	fTuf1	CACATTGACCACGGTAAAAC	941–964	Schneider et al., 1997 [36]
	rTuf1	CCACCTTCACGAATAGAGAAC		
*secA*	secAfor1	GARATGAAAACTGGRGAAGG	713–734	Hodgetts et al., 2008 [37]
	secArev3	GTTTTRGCAGTTCCTGTCATNCC		
*dnaK*	YudnaKf	TGCTCTTTCTTATGGCGTTGA	1135–1164	Yu et al., 2017 [32]
	YudnaKr	CATTGCGATTCCTTGAGATTC		
*rpoB*	YurpoBf	TTCCCACTACGGCAGATTATG	1093–1118	Yu et al., 2017 [32]
	YurpoBr	TGGACGATGCCTCCTTCAC		
*pyrG*	YupyrGf	CCTGGAACAATGAGCCCTTA	1038–1055	Yu et al., 2017 [32]
	YupyrGr	TGGCACGAATAAGAACCTAA		
*gyrB*	YugyrBf	TATTCACCCCAAAACAGG	1248–1279	Yu et al., 2017 [32]
	YugyrBr	AGTAAAGTTCTTATGTGGGC		
*ipt*	Iptf-BamHI	CGGGATCCATGAAAAAAGTAATCGCTAT	739–779	Hu et al., 2013 [38]
	iptr-SalI	ACGCGTCGACATCAGTTTTAAAAAATCGT		

**Table 2:** Genetic parameters calculated for 16S rRNA and each housekeeping gene sequences of PaWB phytoplasma strains by MLST scheme.

Genes	16S rRNA	*rp*	*fusA*	*secY*	*tuf*	*secA*	*dnaK*	*rpoB*	*pyrG*	*gyrB*	*ipt*
No. of strains	122	138	133	134	133	132	130	127	105	86	83
Fragment length (bp)	1252	956	735	1138	782	757	979	955	867	1113	749
cG + C (%)	47.0	34.7	37.5	31.8	37.7	34.7	33.9	33.7	33.0	32.4	30.3
Sequence similarity (%)	99.44	99.58	99.73	99.47	99.62	99.47	99.18	99.06	99.42	99.19	99.73
No. of variable sites	10	1	3	9	4	5	11	14	9	10	4
Percentage of variable sites (%)	0.80	0.10	0.41	0.79	0.51	0.66	1.12	1.47	1.04	0.90	0.53
No. of haplotypes	17	2	3	7	5	4	7	11	10	5	5
Haplotype diversity	0.84	0.08	0.24	0.30	0.25	0.25	0.27	0.29	0.37	0.25	0.30
Nucleotide diversity	0.00128	0.00009	0.00063	0.00090	0.00036	0.00094	0.00163	0.00166	0.00120	0.00137	0.00046
*K_a_*/*K_s_* ratio ^a^	0.35	0	0.29	0.13	2.63	0.54	0.11	1.72	0.39	*	0.14
Tajima’s D	−0.342 ns	−0.581 ns	−0.264 ns	−0.900 ns	−1.166 ns	−0.462 ns	−0.526 ns	−1.022 ns	−0.975 ns	−0.610 ns	−1.178 ns
Fu and Li’s D	−2.269 ns	0.473 ns	−0.649 ns	−1.838 ns	−2.812 ^b^	−1.182 ns	−0.666 ns	−1.420 ns	−1.711 ns	−0.676 ns	−0.222 ns
Fu and Li’s F	−1.888 ns	0.177 ns	−0.619 ns	−1.794 ns	−2.683 ^b^	−1.114 ns	−0.734 ns	−1.520 ns	−1.727 ns	−0.775 ns	0.618 ns
*phi*-test	0.773	/	1.0	1.0	/	1.0	0.042 ^b^	1.0	1.0	1.0	1.0

a: Nonsynonymous to synonymous substitution ratio, determining whether genes are affected by selection pressure during evolution; *: The *K_a_* value of *gyrB* gene was 0.00175, and the *K_s_* value of *gyrB* gene was 0, the *K_a_*/*K_s_* ratio could not be calculated, no statistical significance. b: *p* < 0.05, statistical significance; ns: *p* > 0.05, no statistical significance; /: too few informative characters to use the *phi*-test as implemented.

**Table 3:** Genetic diversity analysis of multi genes in different populations.

Populations	No. of Strains	No. of Variable Sites	MLST STs	Diversity of STs	Nucleotide Diversity	Tajima’s D	Fu and Li’s D	Fu and Li’s F
Northwest China	15	29	ST1, ST2, ST3, ST4, ST5, ST6, ST8	0.724	0.00073	−2.030 ^a^	−2.604 ^a^	−2.814 ^b^
Huang-huai-hai Plain	25	11	ST1, ST4, ST11, ST12, ST14, ST15, ST16, ST17, ST18	0.547	0.00014	−2.341 ^c^	−3.775 ^b^	−3.900 ^b^
Southwest China	19	0	ST1	0	0	/	/	/
South of the Yangtze River	46	32	ST1, ST7, ST9, ST10, ST13, ST19, ST20, ST21, ST22	0.500	0.00153	1.089 ns	−0.137 ns	0.349 ns
Total	105	42	22	0.474	0.0009	−0.914 ns	−1.792 ns	−1.724 ns

a: *p* < 0.05, statistical significance; b: *p* < 0.02, statistical significance; c: *p* < 0.01, statistical significance; ns: *p* > 0.1, no statistical significance; /: uncountable because of no polymorphism.

**Table 4:** Genetic differentiation index (*Fst*) of different populations *.

Population	Northwest China	Huang-Huai-Hai Plain	Southwest China	South of the Yangtze River
Northwest China	0			
Huang-huai-hai Plain	0.01315	0		
Southwest China	0.03968	0	0	
South of the Yangtze River	0.07562	0.20514	0.23148	0

* 0 < *Fst* < 0.05, moderate genetic differentiation among populations; 0.05 < *Fst* < 0.15, large genetic differentiation among populations; *Fst* > 0.25, significant genetic differentiation among populations.

**Table 5:** Gene flow index (*Nm*) of different populations.

Population	Northwest China	Huang-Huai-Hai Plain	Southwest China	South of the Yangtze River
Northwest China	0			
Huang-huai-hai Plain	37.53	0		
Southwest China	12.10	/	0	
South of the Yangtze River	6.11	1.94	1.66	0

## Data Availability

The DNA sequences of the assessed genes are available in the GenBank database, and the accession numbers are given in the paper. All other relevant data are provided within the paper.

## Supplementary Materials

The following supporting information can be downloaded at: https://www.mdpi.com/article/10.3390/biology11111611/s1, Table S1. Paulownia witches’-broom phytoplasma strains source and gene sequencing information; Table S2. Allelic profiles and STs based on combination of the 7 housekeeping genes of 105 PaWB strains; Table S3. Information of all reference phytoplasma strains employed in the study; Table S4. polymorphic nucleotide sites of 16S rRNA gene; Table S5. polymorphic nucleotide sites of *rp* gene; Table S6. polymorphic nucleotide sites of *fusA* gene; Table S7. polymorphic nucleotide sites of *secY* gene; Table S8. polymorphic nucleotide sites of *tuf* gene; Table S9. polymorphic nucleotide sites of *secA* gene; Table S10. polymorphic nucleotide sites of *dnaK* gene; Table S11. polymorphic nucleotide sites of *rpoB* gene; Table S12. polymorphic nucleotide sites of *pyrG* gene; Table S13. polymorphic nucleotide sites of *gyrB* gene; Table S14. polymorphic nucleotide sites of *ipt* gene; Figure S1: Phylogenetic tree constructed based on the concatenate sequences of the 105 PaWB phytoplasma strains and 6 kinds of phytoplasma reference strains employing the maximum-likelihood method; Figure S2: Maximum-likelihood phylogenetic tree for the 22 STs of PaWB phytoplasmas based on the concatenated sequences of six proteins (except ribosomal protein). The scalebar length represents inferred character-state changes under the best-fitting LG + G+I model; Figure S3: Phylogenetic tree constructed based on the *rp* gene of the PaWB phytoplasma strains and 6 kinds of phytoplasma reference strains employing the maximum-likelihood method; Figure S4: Phylogenetic tree constructed based on the *fusA* gene of the PaWB phytoplasma strains and 6 kinds of phytoplasma reference strains employing the maximum-likelihood method; Figure S5: Phylogenetic tree constructed based on the *secY* gene of the PaWB phytoplasma strains and 6 kinds of phytoplasma reference strains employing the maximum-likelihood method; Figure S6: Phylogenetic tree constructed based on the *tuf* gene of the PaWB phytoplasma strains and 6 kinds of phytoplasma reference strains employing the maximum-likelihood method; Figure S7: Phylogenetic tree constructed based on the *secA* gene of the PaWB phytoplasma strains and 6 kinds of phytoplasma reference strains employing the maximum-likelihood method; Figure S8: Phylogenetic tree constructed based on the *dnaK* gene of the PaWB phytoplasma strains and 6 kinds of phytoplasma reference strains employing the maximum-likelihood method; Figure S9: Phylogenetic tree constructed based on the *rpoB* gene of the PaWB phytoplasma strains and 6 kinds of phytoplasma reference strains employing the maximum-likelihood method; Figure S10: Phylogenetic tree constructed based on the *pyrG* gene of the PaWB phytoplasma strains and 6 kinds of phytoplasma reference strains employing the maximum-likelihood method; Figure S11: Phylogenetic tree constructed based on the *gyrB* gene of the PaWB phytoplasma strains and 6 kinds of phytoplasma reference strains employing the maximum-likelihood method; Figure S12: Phylogenetic tree constructed based on the *ipt* gene of the PaWB phytoplasma strains and 6 kinds of phytoplasma reference strains employing the maximum-likelihood method.
