# Supplementary material for: Molecular Diversity and Evolutionary Relatedness of Paulownia Witches’-Broom Phytoplasma in Different Geographical Distributions in China
Source: Biology (Basel). 2022 Nov 3;11(11):1611. doi: 10.3390/biology11111611 (PMC9687790; doi:10.3390/biology11111611)
Supplement: Supplementary file 1 [file biology-11-01611-s001.zip › biology-1909481-supplementary.pdf]

Supplementary material

Table S1. Paulownia witches'-broom phytoplasma strains source and gene sequencing information

Site	Sample codes	Source area	Year	16SrDNA	*rp*	*fusA*	*secY*	*tuf*	*dnaK*	*secA*	*rpoB*	*pyrG*	*gyrB*	*ipt*
1	1-PaWB-JSNJ8	Nanjing, Jiangsu	2014	OP107398	OP124576	OP124443	OP124050	OP123574	OP124797	OP124184	OP124316	-	-	-
2	11-PaWB-JSZJ	Zhenjiang, Jiangsu	2014	OP107399	OP124577	OP124444	OP124051	OP123575	OP124798	OP124185	OP124317	OP125013	-	-
3	12-PaWB-AHHF	Hefei, Anhui	2014	OP107400	OP124578	OP124445	OP124052	OP123576	OP124799	OP124186	OP124318	-	-	-
4	28-PaWB-BJLKY2	Beijing	2014	OP107401	OP124579	OP124446	OP124053	OP123577	OP124800	OP124187	OP124319	-	-	-
5	30-PaWB-HNPY1	Puyang, Henan	2014	-	OP124580	OP124447	OP124054	OP123578	OP124801	OP124188	OP124320	OP125014	-	-
6	32-PaWB-HNZK	Zhoukou, Henan	2014	OP107462	OP124581	OP124448	OP124055	OP123579	OP124802	OP124189	OP124321	OP125015	-	OP124714
7	45-PaWB-HNPY2	Puyang, Henan	2014	OP107402	OP124582	OP124449	OP124056	OP123580	OP124803	OP124190	OP124322	OP125016	-	OP124715
8	46-PaWB-ZJTZ	Taizhou, Zhejiang	2014	OP107403	OP124583	OP124450	OP124057	OP123581	OP124804	OP124191	OP124323	OP125017	OP124927	OP124716
9	48-PaWB-JXJA	Ji'an, Jiangxi	2014	OP107404	OP124584	OP124451	OP124058	OP123582	OP124805	OP124192	OP124324	-	-	OP124717
10	51-PaWB-HBBD2	Baoding, Hebei	2014	OP107463	OP124585	OP124452	OP124059	OP123583	OP124806	OP124193	OP124325	OP125018	OP124928	OP124718
11	55-PaWB-SDJX	Juxian, Shandong	2014	OP107405	OP124586	OP124453	OP124060	OP123584	OP124807	OP124184	OP124326	OP125019	-	-
12	84-PaWB-FJMFS3	Fuzhou, Fujian	2014	OP107406	OP124587	OP124454	OP124061	OP123585	OP124808	-	-	-	-	-
13	86-PaWB-FJMFS5	Fuzhou, Fujian	2014	-	OP124588	-	OP124062	OP123586	-	-	OP124327	-	-	-
14	87-PaWB-FJMFS6	Fuzhou, Fujian	2014	-	OP124589	-	-	OP123587	-	-	-	-	-	-
15	89-PaWB-JXNC1	Nanchang, Jiangxi	2014	OP107407	OP124590	OP124455	-	OP123588	OP124809	-	OP124328	-	-	-
16	90-PaWB-JXNC2	Nanchang, Jiangxi	2014	-	OP124591	OP124456	OP124063	OP123589	OP124810	OP124194	OP124329	OP125020	OP124929	-
17	91-PaWB-HNCS1	Changsha, Hunan	2014	-	OP124592	OP124457	OP124064	OP123590	-	OP124195	OP124330	-	-	-
18	92-PaWB-HNCS2	Changsha, Hunan	2014	OP107408	OP124593	OP124458	OP124065	OP123591	OP124811	OP124196	-	-	-	-
19	95-PaWB-LNDL	Dalian, Liaoning	2015	OP107409	OP124594	OP124459	OP124066	OP123592	OP124812	OP124197	OP124331	OP125021	OP124930	OP124719
20	96-PaWB-HBBD1	Baoding, Hebei	2015	OP107410	OP124595	OP124460	OP124067	OP123593	OP124813	OP124198	OP124332	OP125022	OP124931	OP124720

Site	Sample codes	Source area	Year	16SrDNA	*rp*	*fusA*	*secY*	*tuf*	*dnaK*	*secA*	*rpoB*	*pyrG*	*gyrB*	*ipt*
21	97-PaWB-GZGY	Guiyang, Guizhou	2015	OP107411	OP124596	OP124461	OP124068	OP123594	OP124814	OP124199	OP124333	OP125023	OP124932	OP124721
22	98-PaWB-HNKF	Kaifeng, Henan	2015	OP107412	OP124576	OP124462	OP124069	OP123595	OP124815	OP124200	-	OP125024	OP124933	OP124722
23	100-PaWB-JSNJ1	Nanjing, Jiangsu	2015	OP107464	-	-	-	-	-	-	-	-	-	-
24	104-PaWB-JSNJ5	Nanjing, Jiangsu	2015	OP107465	OP124597	OP124463	OP124070	OP123596	OP124816	OP124201	OP124334	OP125025	-	OP124723
25	105-PaWB-JSNJ6	Nanjing, Jiangsu	2015	OP107413	OP124598	OP124464	OP124071	OP123597	OP124817	OP124202	OP124335	OP125026	OP124934	OP124724
26	107-PaWB-JSSZ2	Suzhou, Jiangsu	2015	OP107414	OP124599	OP124465	OP124072	OP123598	OP124818	OP124203	OP124336	OP125027	OP124935	OP124725
27	108-PaWB-JSSZ3	Suzhou, Jiangsu	2015	-	OP124600	OP124466	-	OP123599	OP124819	OP124204	OP124337	-	-	-
28	109-PaWB-JSSZ4	Suzhou, Jiangsu	2015	OP107415	OP124601	OP124467	OP124073	OP123600	OP124820	OP124205	OP124338	OP125028	OP124936	-
29	111-PaWB-JSSZ6	Suzhou, Jiangsu	2015	OP107416	OP124602	OP124468	OP124074	OP123601	OP124821	OP124206	OP124339	OP125029	OP124937	OP124726
30	118-PaWB-HNZZ1	Zhengzhou, Henan	2015	OP107466	OP124603	OP124469	OP124075	OP123602	OP124822	OP124207	OP124340	OP125030	-	OP124727
31	119-PaWB-HNZZ4	Zhengzhou, Henan	2015	OP107467	OP124604	OP124470	OP124076	OP123603	-	OP124208	-	OP125031	-	-
32	120-PaWB-HNLY2	Luoyang, Henan	2015	OP107417	OP124605	OP124471	OP124077	OP123604	OP124823	OP124209	OP124341	OP125032	-	-
33	121-PaWB-HNLY3	Luoyang, Henan	2015	OP107418	-	-	-	-	-	-	-	-	-	-
34	122-PaWB-HNLY4	Luoyang, Henan	2015	OP107468	OP124606	OP124472	OP124078	OP123605	OP124824	OP124210	OP124342	OP125033	-	OP124728
35	123-PaWB-SAXWN1	Weinan, Shaanxi	2015	OP107469	OP124607	OP124473	OP124079	OP123606	OP124825	OP124211	OP124343	-	OP124938	OP124729
36	124-PaWB-SAXWN2	Weinan, Shaanxi	2015	-	OP124608	OP124474	-	OP123607	OP124826	OP124212	OP124344	OP125034	OP124939	-
37	126-PaWB-SAXWN4	Weinan, Shaanxi	2015	OP107470	OP124609	OP124475	OP124080	OP123608	OP124827	OP124213	-	OP125035	-	OP124730
38	127-PaWB-SAXXA1	Xi’an, Shaanxi	2015	OP107419	OP124610	OP124476	OP124081	OP123609	OP124828	OP124214	OP124345	OP125036	-	-
39	128-PaWB-SAXXA2	Xi’an, Shaanxi	2015	OP107471	OP124611	OP124477	OP124082	OP123610	OP124829	OP124215	OP124346	OP125037	OP124940	OP124731
40	129-PaWB-SAXXA3	Xi’an, Shaanxi	2015	OP107420	OP124612	OP124478	OP124083	OP123611	OP124830	OP124216	OP124347	OP125038	OP124941	OP124732
41	130-PaWB-SAXXA4	Xi’an, Shaanxi	2015	OP107421	OP124613	OP124479	OP124084	OP123612	OP124831	OP124217	OP124348	OP125039	-	-
42	131-PaWB-SAXXA5	Xi’an, Shaanxi	2015	OP107472	OP124614	OP124480	OP124085	OP123613	OP124832	OP124218	OP124349	-	-	-
43	132-PaWB-SAXXA6	Xi’an, Shaanxi	2015	OP107422	OP124615	OP124481	OP124086	OP123614	OP124833	OP124219	OP124350	OP125040	OP124942	-
44	133-PaWB-SXTY1	Taiyuan, Shanxi	2015	OP107423	OP124616	OP124482	OP124087	OP123615	OP124834	OP124220	OP124351	OP125041	OP124943	OP124733

Site	Sample codes	Source area	Year	16SrDNA	*rp*	*fusA*	*secY*	*tuf*	*dnaK*	*secA*	*rpoB*	*pyrG*	*gyrB*	*ipt*
45	134-PaWB-SXTY3	Taiyuan, Shanxi	2015	OP107424	OP124617	OP124483	OP124088	OP123616	OP124835	OP124221	OP124352	OP125042	OP124944	-
46	135-PaWB-SXTY5	Taiyuan, Shanxi	2015	OP107425	OP124618	OP124484	OP124089	OP123617	OP124836	OP124222	OP124353	OP125043	OP124945	OP124734
47	136-PaWB-SXTY6	Taiyuan, Shanxi	2015	OP107426	OP124619	OP124485	OP124090	OP123618	OP124837	OP124223	OP124354	OP125044	-	-
48	137-PaWB-SXTY7	Taiyuan, Shanxi	2015	OP107427	OP124620	OP124486	OP124091	OP123619	OP124838	OP124224	OP124355	OP125045	-	OP124735
49	138-PaWB-SXTY8	Taiyuan, Shanxi	2015	OP107428	OP124621	OP124487	OP124092	OP123620	OP124839	OP124225	OP124356	OP125046	-	OP124736
50	139-PaWB-SXTY9	Taiyuan, Shanxi	2015	OP107473	OP124622	OP124488	OP124093	OP123621	OP124840	OP124226	OP124357	OP125047	OP124946	OP124737
51	140-PaWB-HBJM1	Jinmen, Hubei	2015	OP107474	OP124623	OP124489	OP124094	OP123622	OP124841	OP124227	OP124358	OP125048	-	-
52	141-PaWB-HBJM2	Jinmen, Hubei	2015	OP107429	OP124624	OP124490	OP124095	OP123623	OP124842	OP124228	OP124359	OP125049	-	-
53	142-PaWB-HBJM3	Jinmen, Hubei	2015	OP107430	OP124625	-	OP124096	OP123624	OP124843	OP124229	OP124360	OP125050	OP124947	OP124738
54	144-PaWB-AHBZ2	Bozhou, Anhui	2015	OP107475	OP124626	OP124491	OP124097	OP123625	OP124844	OP124230	OP124361	OP125051	OP124948	-
55	145-PaWB-AHBZ3	Bozhou, Anhui	2015	OP107431	OP124627	OP124492	OP124098	OP123626	OP124845	OP124231	OP124362	OP125052	OP124949	OP124739
56	147-PaWB-SAXYA	Yan’an, Shaanxi	2015	OP107476	OP124628	OP124493	OP124099	OP123627	OP124846	OP124232	OP124363	-	-	-
57	192-PaWB-FJFZ1	Fuzhou, Fujian	2015	OP107432	OP124629	OP124494	-	-	OP124847	OP124233	-	-	-	-
58	194-PaWB-FJFZ3	Fuzhou, Fujian	2015	OP107477	OP124630	OP124495	OP124100	OP123628	OP124848	OP124234	OP124364	OP125053	-	-
59	195-PaWB-FJFZ4	Fuzhou, Fujian	2015	OP107433	OP124631	OP124496	OP124101	OP123629	OP124849	OP124235	OP124365	OP125054	OP124950	OP124740
60	238-PaWB-HBLS1	Lingshou, Hebei	2015	OP107478	OP124632	OP124497	OP124102	OP123630	OP124850	OP124236	OP124366	OP125055	OP124951	-
61	239-PaWB-HBLS2	Lingshou, Hebei	2015	OP107434	OP124633	OP124498	OP124103	OP123631	OP124851	OP124237	OP124367	OP125056	OP124952	OP124741
62	240-PaWB-HBLS3	Lingshou, Hebei	2015	OP107435	OP124634	OP124499	OP124104	OP123632	OP124852	OP124238	OP124368	OP125057	OP124953	OP124742
63	246-PaWB-SDJN1	Jinan, Shandong	2015	OP107436	OP124635	OP124500	OP124105	OP123633	OP124853	OP124239	OP124369	OP125058	OP124954	OP124743
64	247-PaWB-SDJN2	Jinan, Shandong	2015	OP107479	OP124636	OP124501	OP124106	OP123634	OP124854	OP124240	OP124370	OP125059	OP124955	-
65	249-PaWB-SDJN4	Jinan, Shandong	2015	OP107437	OP124637	OP124502	OP124107	OP123635	OP124855	OP124241	OP124371	OP125060	OP124956	OP124744
66	250-PaWB-ZJWZ1	Wenzhou, Zhejiang	2015	OP107480	OP124638	OP124503	OP124108	OP123636	OP124856	OP124242	OP124372	OP125061	OP124957	OP124745
67	251-PaWB-ZJWZ2	Wenzhou, Zhejiang	2015	OP107438	-	OP124504	OP124109	OP123637	OP124857	-	OP124373	-	OP124958	OP124746
68	252-PaWB-ZJWZ3	Wenzhou, Zhejiang	2015	OP107439	OP124639	OP124505	OP124110	OP123638	OP124858	OP124243	OP124374	OP125062	OP124959	OP124747

Site	Sample codes	Source area	Year	16SrDNA	*rp*	*fusA*	*secY*	*tuf*	*dnaK*	*secA*	*rpoB*	*pyrG*	*gyrB*	*ipt*
69	254-PaWB-ZJWZ5	Wenzhou, Zhejiang	2015	OP107440	OP124640	OP124506	OP124111	OP123639	OP124859	OP124244	OP124375	OP125063	OP124960	OP124748
70	255-PaWB-ZJLS1	Lishui, Zhejiang	2015	OP107441	OP124641	OP124507	OP124112	OP123640	OP124860	OP124245	-	-	-	OP124749
71	256-PaWB-ZJLS2	Lishui, Zhejiang	2015	OP107442	OP124642	OP124508	OP124113	OP123641	OP124861	OP124246	OP124376	OP125064	OP124961	OP124750
72	257-PaWB-ZJLS3	Lishui, Zhejiang	2015	OP107443	OP124643	OP124509	OP124114	OP123642	OP124862	OP124247	OP124377	OP125065	OP124962	OP124751
73	259-PaWB-ZJLS5	Lishui, Zhejiang	2015	OP107481	OP124644	OP124510	OP124115	OP123643	OP124863	OP124248	OP124378	-	-	-
74	260-PaWB-ZJLS6	Lishui, Zhejiang	2015	OP107444	OP124645	OP124511	OP124116	OP123644	OP124864	OP124249	OP124379	OP125066	OP124963	OP124752
75	262-PaWB-ZJJH1	Jinhua, Zhejiang	2015	OP107445	OP124646	OP124512	OP124117	OP123645	OP124865	OP124250	OP124380	-	OP124964	OP124753
76	263-PaWB-ZJJH2	Jinhua, Zhejiang	2015	OP107446	OP124647	OP124513	OP124118	OP123646	OP124866	OP124251	OP124381	OP125067	OP124965	OP124754
77	264-PaWB-ZJJH3	Jinhua, Zhejiang	2015	-	OP124648	OP124514	OP124119	OP123647	OP124867	OP124252	OP124382	OP125068	-	OP124755
78	265-PaWB-ZJJH4	Jinhua, Zhejiang	2015	OP107447	OP124649	OP124515	-	-	OP124868	OP124253	-	-	-	-
79	266-PaWB-ZJJH5	Jinhua, Zhejiang	2015	OP107448	OP124650	OP124516	OP124120	OP123648	OP124869	OP124254	OP124383	OP125069	OP124966	OP124756
80	268-PaWB-ZJJH7	Jinhua, Zhejiang	2015	OP107482	OP124651	-	OP124121	OP123649	OP124870	OP124255	OP124384	OP125070	OP124967	-
81	272-PaWB-ZJHZ1	Hangzhou, Zhejiang	2015	OP107449	OP124652	OP124517	OP124122	OP123650	OP124871	OP124256	OP124385	OP125071	-	OP124757
82	273-PaWB-ZJHZ2	Hangzhou, Zhejiang	2015	OP107450	OP124653	OP124518	OP124123	OP123651	OP124872	OP124257	OP124386	OP125072	OP124968	OP124758
83	274-PaWB-ZJHZ3	Hangzhou, Zhejiang	2015	OP107451	OP124654	OP124519	OP124124	OP123652	OP124873	OP124258	OP124387	OP125073	OP124969	-
84	275-PaWB-ZJHZ4	Hangzhou, Zhejiang	2015	OP107483	OP124655	OP124520	OP124125	OP123653	OP124874	OP124259	OP124388	OP125074	OP124970	OP124759
85	276-PaWB-ZJHZ5	Hangzhou, Zhejiang	2015	OP107484	OP124656	OP124521	OP124126	OP123654	OP124875	OP124260	OP124389	OP125075	OP124971	-
86	277-PaWB-ZJHZ6	Hangzhou, Zhejiang	2015	OP107485	OP124657	OP124522	OP124127	OP123655	OP124876	OP124261	OP124390	OP125076	OP124972	OP124760
87	278-PaWB-ZJHZ7	Hangzhou, Zhejiang	2015	OP107486	OP124658	OP124523	OP124128	OP123656	OP124877	-	OP124391	-	-	-
88	279-PaWB-ZJCA1	Chun’an, Zhejiang	2015	OP107487	OP124659	OP124524	OP124129	OP123657	OP124878	OP124262	-	OP125077	OP124973	OP124761
89	280-PaWB-ZJCA2	Chun’an, Zhejiang	2015	OP107488	OP124660	OP124525	OP124130	OP123658	OP124879	OP124263	OP124392	OP125078	-	OP124762
90	281-PaWB-ZJCA3	Chun’an, Zhejiang	2015	OP107489	OP124661	OP124526	OP124131	OP123659	OP124880	OP124264	OP124393	OP125079	OP124974	OP124763
91	282-PaWB-ZJCA4	Chun’an, Zhejiang	2015	OP107452	OP124662	OP124527	OP124132	OP123660	OP124881	OP124265	OP124394	-	-	OP124764
92	283-PaWB-ZJCA5	Chun’an, Zhejiang	2015	OP107453	OP124663	OP124528	OP124133	OP123661	OP124882	OP124266	OP124395	OP125080	-	-

Site	Sample codes	Source area	Year	16SrDNA	*rp*	*fusA*	*secY*	*tuf*	*dnaK*	*secA*	*rpoB*	*pyrG*	*gyrB*	*ipt*
93	284-PaWB-ZJCA6	Chun’an, Zhejiang	2015	OP107490	OP124664	OP124529	OP124134	OP123662	OP124883	OP124267	OP124396	OP125081	OP124975	OP124765
94	285-PaWB-ZJCA7	Chun’an, Zhejiang	2015	-	OP124665	OP124530	OP124135	OP123663	OP124884	OP124268	OP124397	-	OP124976	OP124766
95	286-PaWB-ZJCA8	Chun’an, Zhejiang	2015	-	OP124666	OP124531	OP124136	OP123664	OP124885	OP124269	OP124398	-	OP124977	OP124767
96	289-PaWB-HBTX4	Tangxian, Hebei	2015	-	OP124667	OP124532	OP124137	OP123665	OP124886	OP124270	OP124399	-	OP124978	-
97	290-PaWB-HBTX3	Tangxian, Hebei	2015	OP107454	OP124668	OP124533	OP124138	OP123666	OP124887	OP124271	OP124400	OP125082	OP124979	OP124768
98	291-PaWB-HBTX2	Tangxian, Hebei	2015	OP107491	OP124669	OP124534	OP124139	OP123667	-	OP124272	OP124401	OP125083	OP124980	OP124769
99	292-PaWB-HBTX1	Tangxian, Hebei	2015	OP107492	OP124670	OP124535	OP124140	OP123668	OP124888	OP124273	OP124402	OP125084	OP124981	OP124770
100	297-PaWB-BJ3	Beijing	2015	OP107493	OP124671	OP124536	OP124141	OP123669	OP124889	OP124274	OP124403	-	OP124982	OP124771
101	298-PaWB-BJ4	Beijing	2015	OP107494	OP124672	OP124537	OP124142	OP123670	OP124890	OP124275	OP124404	OP125085	OP124983	OP124772
102	299-PaWB-BJ5	Beijing	2015	OP107455	OP124673	OP124538	OP124143	OP123671	OP124891	OP124276	OP124405	OP125086	OP124984	OP124773
103	300-PaWB-BJ6	Beijing	2015	OP107456	OP124674	OP124539	OP124144	OP123672	OP124892	OP124277	OP124406	OP125087	OP124985	-
104	301-PaWB-BJ7	Beijing	2015	OP107495	OP124675	OP124540	OP124145	OP123673	OP124893	OP124278	OP124407	OP125088	OP124986	OP124774
105	302-PaWB-BJ8	Beijing	2015	OP107457	OP124676	OP124541	OP124146	OP123674	OP124894	OP124279	OP124408	-	OP124987	OP124775
106	303-PaWB-BJ1	Beijing	2015	-	OP124677	OP124542	OP124147	OP123675	OP124895	OP124280	OP124409	OP125089	OP124988	-
107	359-PaWB-CQ7	Chongqing	2015	OP107496	OP124678	OP124543	OP124148	OP123676	OP124896	OP124281	OP124410	-	OP124989	-
108	373-PaWB-SCCD3	Chengdu, Sichaun	2015	OP107497	OP124679	OP124544	OP124149	OP123677	OP124897	OP124282	OP124411	OP125090	-	-
109	374-PaWB-SCCD4	Chengdu, Sichuan	2015	OP107498	OP124680	OP124545	OP124150	OP123678	OP124898	OP124283	OP124412	OP125091	OP124990	-
110	375-PaWB-SCLS1	Leshan, Sichuan	2015	OP107458	OP124681	OP124546	OP124151	OP123679	OP124899	OP124284	OP124413	OP125092	-	-
111	376-PaWB-SCLS2	Leshan, Sichuan	2015	OP107499	OP124682	OP124547	OP124152	OP123680	OP124900	OP124285	OP124414	OP125093	-	OP124776
112	377-PaWB-CQ1	Chongqing	2015	OP107459	OP124683	OP124548	OP124153	OP123681	OP124901	OP124286	OP124415	-	-	-
113	381-PaWB-CQ2	Chongqing	2015	OP107500	OP124684	OP124549	OP124154	OP123682	OP124902	OP124287	OP124416	OP125094	OP124991	-
114	383-PaWB-CQ4	Chongqing	2015	OP107460	OP124685	OP124550	OP124155	-	OP124903	OP124288	OP124417	-	OP124992	-
115	403-PaWB-SCCD1-2	Chengdu, Sichuan	2015	OP107461	OP124686	OP124551	OP124156	OP123683	OP124904	OP124289	OP124418	OP125095	OP124993	OP124777
116	407-PaWB-HBGG1	Wuhan, Hubei	2016	-	OP124689	-	OP124157	-	-	OP124290	OP124419	-	OP124994	-

Site	Sample codes	Source area	Year	16SrDNA	*rp*	*fusA*	*secY*	*tuf*	*dnaK*	*secA*	*rpoB*	*pyrG*	*gyrB*	*ipt*
117	408-PaWB-HBGG2	Wuhan, Hubei	2016	-	OP124688	OP124553	OP124158	OP123684	-	OP124291	OP124420	-	-	-
118	409-PaWB-JXGF1	Guangfeng, Jiangxi	2016	-	OP124687	OP124552	OP124159	-	-	-	-	-	-	-
119	410-PaWB-FJFZ73	Fuzhou, Fujian	2019	OP107501	OP124690	OP124554	OP124160	OP123685	OP124905	OP124292	OP124421	OP125096	OP124995	OP124778
120	411-PaWB-FJFZ79	Fuzhou, Fujian	2019	OP107502	OP124691	OP124555	OP124161	OP123686	OP124906	OP124293	OP124422	OP125097	OP124996	OP124779
121	412-PaWB-FJFZ80	Fuzhou, Fujian	2019	OP107503	OP124692	OP124556	OP124162	OP123687	OP124907	OP124294	OP124423	OP125098	OP124997	OP124780
122	413-PaWB-FJFZ81	Fuzhou, Fujian	2019	OP107504	OP124693	OP124557	OP124163	OP123688	OP124908	OP124295	OP124424	OP125099	OP124998	OP124781
123	414-PaWB-FJFZ84	Fuzhou, Fujian	2019	OP107505	OP124694	OP124558	OP124164	OP123689	OP124909	OP124296	OP124425	OP125100	OP124999	OP124782
124	415-PaWB-FJFZ85	Fuzhou, Fujian	2019	-	OP124695	OP124559	OP124165	OP123690	OP124910	OP124297	OP124426	OP125101	-	OP124783
125	416-PaWB-FJFZ86	Fuzhou, Fujian	2019	-	OP124696	OP124560	OP124166	OP123691	OP124911	OP124298	OP124427	OP125102	-	OP124784
126	417-PaWB-FJNP89	Nanping, Fujian	2019	OP107506	OP124697	OP124561	OP124167	OP123692	OP124912	OP124299	OP124428	OP125103	OP125000	OP124785
127	418-PaWB-FJNP95	Nanping, Fujian	2019	OP107507	OP124698	OP124562	OP124168	OP123693	OP124913	OP124300	OP124429	OP125104	OP125001	OP124786
128	420-PaWB-SCCD5a	Chengdu, Sichuan	2019	OP107508	OP124699	OP124563	OP124169	OP123694	OP124914	OP124301	OP124430	OP125105	OP125002	OP124787
129	421-PaWB-GZGY1	Guiyang, Guizhou	2019	OP107509	OP124700	OP124564	OP124170	OP123695	OP124915	OP124302	OP124431	OP125106	OP125003	OP124788
130	422-PaWB-GZGY2	Guiyang, Guizhou	2019	OP107510	OP124701	OP124565	OP124171	OP123696	OP124916	OP124303	OP124432	OP125107	OP125004	OP124789
131	423-PaWB-GZGY3	Guiyang, Guizhou	2019	OP107511	OP124702	OP124566	OP124172	OP123697	OP124917	OP124304	OP124433	OP125108	OP125005	OP124790
132	424-PaWB-GZZY2	Zunyi, Guizhou	2019	OP107512	OP124703	OP124567	OP124173	OP123698	OP124918	OP124305	OP124434	OP125109	OP125006	OP124791
133	425-PaWB-GZZY3	Zunyi, Guizhou	2019	OP107513	OP124704	OP124568	OP124174	OP123699	OP124919	OP124306	OP124435	OP125110	OP125007	OP124792
134	426-PaWB-YNKMd	Kunming, Yunnan	2019	OP107514	OP124705	OP124569	OP124175	OP123700	OP124920	OP124307	OP124436	OP125111	OP125008	OP124793
135	427-PaWB-YNKM2	Kunming, Yunnan	2019	OP107515	OP124706	OP124570	OP124176	OP123701	OP124921	OP124308	OP124437	OP125112	OP125009	OP124794
136	428-PaWB-YNKM3	Kunming, Yunnan	2019	OP107516	OP124707	OP124571	OP124177	OP123702	OP124922	OP124309	OP124438	OP125113	OP125010	OP124795
137	429-PaWB-YNKM4	Kunming, Yunnan	2019	OP107517	OP124708	OP124572	OP124178	OP123703	OP124923	OP124310	OP124439	OP125114	OP125011	OP124796
138	430-PaWB-JXGF	Guangfeng, Jiangxi	2019	OP107518	OP124709	OP124573	OP124179	OP123704	OP124924	OP124311	OP124440	OP125115	-	-
139	431-PaWB-JXGFhf	Guangfeng, Jiangxi	2019	-	OP124710	OP124574	OP124180	OP123705	OP124925	OP124312	OP124441	OP125116	-	-
140	432-PaWB-JXFY1	Fenyi, Jiangxi	2019	OP107519	OP124711	OP124575	OP124181	OP123706	OP124926	OP124313	OP124442	OP125117	OP125012	-
141	433-PaWB-SAXWN34	Weinan, Shaanxi	2018	-	OP124712	-	OP124182	-	-	OP124314	-	-	-	-

Site	Sample codes	Source area	Year	16SrDNA	*rp*	*fusA*	*secY*	*tuf*	*dnaK*	*secA*	*rpoB*	*pyrG*	*gyrB*	*ipt*
142	434-PaWB-SAXXA40	Xi’an, Shaanxi	2018	-	OP124713	-	OP124183	-	-	OP124315	-	-	-	-

Note: “-” means didn’t have sequences

Table S2. Allelic profiles and STs based on combination of the 7 housekeeping genes of 105 PaWB strains

STs	No. of strains	Housekeeping gene haplotype							Allelic profiles
		H_{dnaK}	H_{fusA}	H_{rp}	H_{rpoB}	H_{secA}	H_{secY}	H_{tuf}	
ST1	76	1	1	1	1	1	1	1	1-1-1-1-1-1-1
ST2	1	1	1	1	1	1	4	1	1-1-1-1-1-4-1
ST3	1	2	2	2	5	1	2	2	2-2-2-5-1-2-2
ST4	3	1	1	1	1	2	1	1	1-1-1-1-2-1-1
ST5	1	1	1	1	6	2	1	1	1-1-1-6-2-1-1
ST6	1	1	1	1	1	1	5	1	1-1-1-1-1-5-1
ST7	1	5	1	1	1	1	1	1	5-1-1-1-1-1-1
ST8	1	1	1	1	7	1	5	1	1-1-1-7-1-5-1
ST9	1	2	2	2	8	1	6	2	2-2-2-8-1-6-2
ST10	1	2	2	2	3	1	2	2	2-2-2-3-1-2-2
ST11	1	1	1	1	1	1	1	3	1-1-1-1-1-1-3
ST12	1	1	1	1	1	3	1	1	1-1-1-1-3-1-1
ST13	1	1	1	1	1	1	7	1	1-1-1-1-1-7-1
ST14	1	1	3	1	1	1	1	1	1-3-1-1-1-1-1
ST15	1	1	1	1	1	1	1	4	1-1-1-1-1-1-4
ST16	1	3	1	1	1	1	1	1	3-1-1-1-1-1-1
ST17	1	1	1	1	2	1	1	1	1-1-1-2-1-1-1
ST18	1	1	1	1	1	1	3	1	1-1-1-1-1-3-1
ST19	7	2	2	1	3	2	2	2	2-2-1-3-2-2-2
ST20	1	2	2	1	10	2	2	2	2-2-1-10-2-2-2
ST21	1	2	2	2	5	2	2	5	2-2-2-5-2-2-5
ST22	1	2	2	1	11	2	2	2	2-2-1-11-2-2-2

Table S3. Information of all reference phytoplasma strains employed in the study

Isolates	Causing disease	Collected regions	Group	GenBank accession numbers										
				16S	*rp*	*fusA*	*secY*	*tuf*	*dnaK*	*secA*	*rpoB*	*pyrG*	*gyrB*	*ipt*
AYWB	Aster yellows witches'-broom	U.S.A.	16SrI	CP000061	CP000061	CP000061	CP000061	CP000061	CP000061	CP000061	CP000061	CP000061	CP000061	CP000061
OY-M	Onion yellows	Japan	16SrI	AP006628	AP006628	AP006628	AP006628	AP006628	AP006628	AP006628	AP006628	AP006628	AP006628	AP006628
LY-fjsm1	Lettuce yellows	Sanming, Fujian, China	16SrI	KP662129	KP662147	KP662255	KP662201	KP662165	KP662237	KP662183	KP662309	KP662291	KP662273	KP662219
LY-fjsm2	Lettuce yellows	Sanming, Fujian, China	16SrI	KP662130	KP662148	KP662256	KP662202	KP662166	KP662238	KP662184	KP662310	KP662292	KP662274	KP662220
PeV-hnhk	Periwinkle virescence	Haikou, Hainan, China	16SrI	KP662136	KP662154	KP662262	KP662208	KP662172	KP662244	KP662190	KP662316	KP662298	KP662280	KP662226
CWB-fjya	Chinaberry witches'-broom	Yong'an, Fujian, China	16SrI	KP662124	KP662142	KP662250	KP662196	KP662160	KP662232	KP662178	KP662304	KP662286	KP662268	KP662214
CWB-jxnc	Chinaberry witches'-broom	Nanchang, Jiangxi, China	16SrI	KP662126	KP662144	KP662252	KP662198	KP662162	KP662234	KP662180	KP662306	KP662288	KP662270	KP662216
MD-ahhf	Mulberry dwarf	Hefei, Anhui, China	16SrI	KP662131	KP662149	KP662257	KP662203	KP662167	KP662239	KP662185	KP662311	KP662293	KP662275	KP662221
MD-zjca	Mulberry dwarf	Chun'an Zhejiang, China	16SrI	KP662132	KP662150	KP662258	KP662204	KP662168	KP662240	KP662186	KP662312	KP662294	KP662276	KP662222
PaWB-Zhengzhou	Paulownia witches'-broom	Zhengzhou, Henan, China	16SrI	CP066882	CP066882	CP066882	CP066882	CP066882	CP066882	CP066882	CP066882	CP066882	CP066882	CP066882

Table S4. polymorphic nucleotide sites of 16S rRNA gene

16S rRNA (no. of strains)	Polymorphic Nucleotide Sites									
	63	82	86	118	212	300	450	559	685	724
$H_{16S\ rRNA}$1(37)	A	G	A	A	G	G	A	G	G	C
$H_{16S\ rRNA}$2(22)	•	•	•	•	•	•	G	•	T	•
$H_{16S\ rRNA}$3(19)	•	•	•	•	•	•	G	•	•	•
$H_{16S\ rRNA}$4(12)	•	•	•	•	•	•	•	•	T	•
$H_{16S\ rRNA}$5(8)	•	•	G	•	•	A	•	•	•	•
$H_{16S\ rRNA}$6(8)	•	T	•	•	•	•	•	•	•	•
$H_{16S\ rRNA}$7(5)	•	T	•	•	•	•	•	•	T	•
$H_{16S\ rRNA}$8(2)	•	•	•	•	•	A	•	•	•	•
$H_{16S\ rRNA}$9(1)	•	•	•	•	•	A	•	•	T	•
$H_{16S\ rRNA}$10(1)	•	•	•	•	•	A	G	•	•	•
$H_{16S\ rRNA}$11(1)	•	•	•	•	•	•	•	•	T	T
$H_{16S\ rRNA}$12(1)	•	•	•	G	•	•	G	•	T	•
$H_{16S\ rRNA}$13(1)	•	•	G	•	•	A	•	T	•	•
$H_{16S\ rRNA}$14(1)	•	•	G	•	•	•	G	•	•	•
$H_{16S\ rRNA}$15(1)	•	•	G	•	T	A	•	•	•	•
$H_{16S\ rRNA}$16(1)	G	•	•	•	•	•	G	•	•	•
$H_{16S\ rRNA}$17(1)	•	•	G	•	•	A	G	•	T	•

Additional mutations that were found in regions for which not all genotypes were sequenced were omitted from this comparison.

The numbers of polymorphic nucleotide sites refer to variable position observed in final alignment.

Table S5. polymorphic nucleotide sites of *rp* gene

rp (no. of strains)	Polymorphic Nucleotide Sites			
	272	273	274	597
H_{rp}1(132)	A	C	C	G
H_{rp}2(6)	/	/	/	A

Additional mutations that were found in regions for which not all genotypes were sequenced were omitted from this comparison.

The numbers of polymorphic nucleotide sites refer to variable position observed in final alignment.

Table S6. polymorphic nucleotide sites of *fusA* gene

fusA (no. of strains)	Polymorphic Nucleotide Sites		
	85	114	436
H_{fusA}1(115)	G	C	A
H_{fusA}2(17)	C	A	•
H_{fusA}3(1)	•	•	G

Additional mutations that were found in regions for which not all genotypes were sequenced were omitted from this comparison.

The numbers of polymorphic nucleotide sites refer to variable position observed in final alignment.

Table S7. polymorphic nucleotide sites of *secY* gene

secY (no. of str ains)	Polymorphic Nucleotide Sites								
	18	28	36	87	130	438	712	802	922
$H_{secY}1$(111)	T	A	G	G	T	T	G	A	A
$H_{secY}2$(17)	•	•	•	•	C	•	A	G	T
$H_{secY}3$(1)	•	G	•	•	•	•	•	•	•
$H_{secY}4$(1)	•	•	•	•	•	A	•	•	•
$H_{secY}5$(2)	G	•	•	•	•	•	•	•	•
$H_{secY}6$(1)	•	•	•	C	C	•	A	G	T
$H_{secY}7$(1)	•	•	C	•	•	•	•	•	•

Additional mutations that were found in regions for which not all genotypes were sequenced were omitted from this comparison. The numbers of polymorphic nucleotide sites refer to variable position observed in final alignment.

Table S8. polymorphic nucleotide sites of *tuf* gene

tuf (no. of strains)	Polymorphic Nucleotide Sites			
	183	421	528	763
$H_{tuf}1$(114)	T	A	A	G
$H_{tuf}2$(16)	•	G	•	•
$H_{tuf}3$(1)	•	G	C	•
$H_{tuf}4$(1)	•	•	•	C
$H_{tuf}5$(1)	G	•	•	•

Additional mutations that were found in regions for which not all genotypes were sequenced were omitted from this comparison. The numbers of polymorphic nucleotide sites refer to variable position observed in final alignment.

Table S9. polymorphic nucleotide sites of *secA* gene

secA (no. of strains)	Polymorphic Nucleotide Sites				
	38	113	222	405	570
$H_{secA}1$(113)	C	A	G	T	G
$H_{secA}2$(17)	•	C	•	A	A
$H_{secA}3$(1)	•	•	T	•	•
$H_{secA}4$(1)	T	•	•	•	•

Additional mutations that were found in regions for which not all genotypes were sequenced were omitted from this comparison. The numbers of polymorphic nucleotide sites refer to variable position observed in final alignment.

Table S10. polymorphic nucleotide sites of *dnaK* gene

dnaK (no. of strains)	Polymorphic Nucleotide Sites										
	36	164	195	263	477	509	710	962	964	965	974
$H_{dnaK}1$(110)	G	T	C	C	T	A	C	A	A	T	T
$H_{dnaK}2$(15)	A	C	T	T	C	C	•	•	•	A	•
$H_{dnaK}3$(1)	•	•	•	•	•	•	•	G	•	•	•

H_{dnaK}4(1)	•	•	•	•	•	•	•	•	•	•	C
H_{dnaK}5(1)	•	•	•	•	•	•	•	•	G	•	•
H_{dnaK}6(1)	A	C	T	T	C	C	•	•	•	A	C
H_{dnaK}7(1)	•	•	•	•	•	•	G	•	•	•	•

Additional mutations that were found in regions for which not all genotypes were sequenced were omitted from this comparison.

The numbers of polymorphic nucleotide sites refer to variable position observed in final alignment.

Table S11. polymorphic nucleotide sites of *rpoB* gene

rpoB (no. of	Polymorphic Nucleotide Sites													
strains)	9	59	78	79	242	389	432	527	534	672	706	753	877	922
H_{rpoB}1(107)	A	T	C	C	T	G	T	A	C	G	C	G	C	A
H_{rpoB}2(1)	•	•	•	•	•	•	•	•	•	•	•	A	•	•
H_{rpoB}3(9)	•	G	A	•	•	•	C	G	T	•	T	A	•	•
H_{rpoB}4(1)	•	G	•	•	A	•	C	G	T	•	T	A	A	•
H_{rpoB}5(2)	•	G	•	•	•	•	C	G	T	•	T	A	A	•
H_{rpoB}6(2)	G	•	•	•	•	•	•	•	•	•	•	•	•	•
H_{rpoB}7(1)	•	•	•	•	•	•	•	•	•	•	•	•	•	G
H_{rpoB}8(1)	•	G	A	•	•	•	C	G	T	C	T	A	•	•
H_{rpoB}9(1)	•	•	•	•	•	T	•	•	•	•	•	•	•	•
H_{rpoB}10(1)	•	G	A	A	•	•	C	G	T	•	T	A	•	•
H_{rpoB}11(1)	•	G	•	•	•	•	C	G	T	•	T	A	•	•

Additional mutations that were found in regions for which not all genotypes were sequenced were omitted from this comparison.

The numbers of polymorphic nucleotide sites refer to variable position observed in final alignment.

Table S12. polymorphic nucleotide sites of *pyrG* gene

pyrG (no. of strains)	Polymorphic Nucleotide Sites								
	39	67	222	426	499	554	582	696	769
H_{pyrG}1(83)	A	A	C	C	C	C	C	C	G
H_{pyrG}2(1)	•	•	•	•	•	T	•	•	•
H_{pyrG}3(1)	•	•	•	•	•	•	•	•	A
H_{pyrG}4(2)	•	G	•	•	•	•	•	•	•
H_{pyrG}5(1)	•	•	•	•	•	•	T	•	•
H_{pyrG}6(1)	C	•	•	•	•	•	•	•	•
H_{pyrG}7(11)	•	•	T	T	•	•	•	A	A
H_{pyrG}8(3)	•	•	•	T	•	•	•	A	A
H_{pyrG}9(1)	•	•	•	•	•	•	•	A	A
H_{pyrG}10(1)	•	•	•	•	A	•	•	•	•

Additional mutations that were found in regions for which not all genotypes were sequenced were omitted from this comparison.

The numbers of polymorphic nucleotide sites refer to variable position observed in final alignment.

Table S13. polymorphic nucleotide sites of *gyrB* gene

gyrB (no. of strains)	Polymorphic Nucleotide Sites									
	37	86	168	269	285	390	464	483	675	1103
$H_{gyrB}1$(74)	T	C	C	A	A	T	G	C	T	A
$H_{gyrB}2$(9)	•	A	A	•	G	C	A	T	C	•
$H_{gyrB}3$(1)	•	•	•	•	•	•	•	•	•	G
$H_{gyrB}4$(1)	A	•	•	•	•	•	•	•	•	•
$H_{gyrB}5$(1)	•	A	A	T	G	C	A	T	C	•

Additional mutations that were found in regions for which not all genotypes were sequenced were omitted from this comparison.

The numbers of polymorphic nucleotide sites refer to variable position observed in final alignment.

Table S14. polymorphic nucleotide sites of *ipt* gene

ipt (no. of strains)	Polymorphic Nucleotide Sites			
	102	158	322	734
$H_{ipt}1$(69)	G	C	C	C
$H_{ipt}2$(2)	•	•	•	T
$H_{ipt}3$(4)	A	•	•	•
$H_{ipt}4$(7)	•	T	•	•
$H_{ipt}5$(1)	•	•	A	•

Additional mutations that were found in regions for which not all genotypes were sequenced were omitted from this comparison.

The numbers of polymorphic nucleotide sites refer to variable position observed in final alignment.

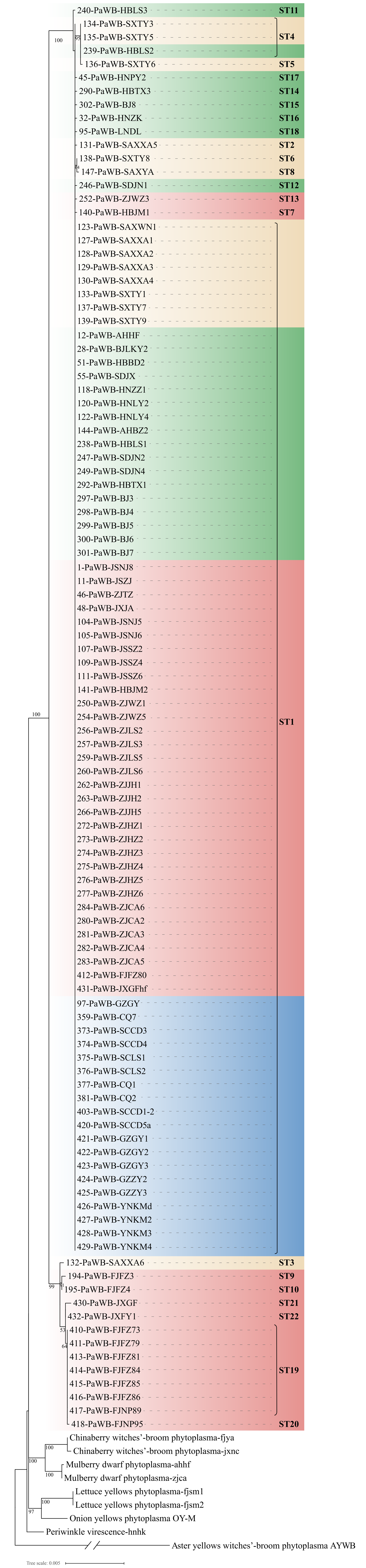

Figure S1. Phylogenetic tree constructed based on the concatenate sequences of the 105 PaWB phytoplasma strains and 6 kinds of phytoplasma reference strains employing the maximum-likelihood method.

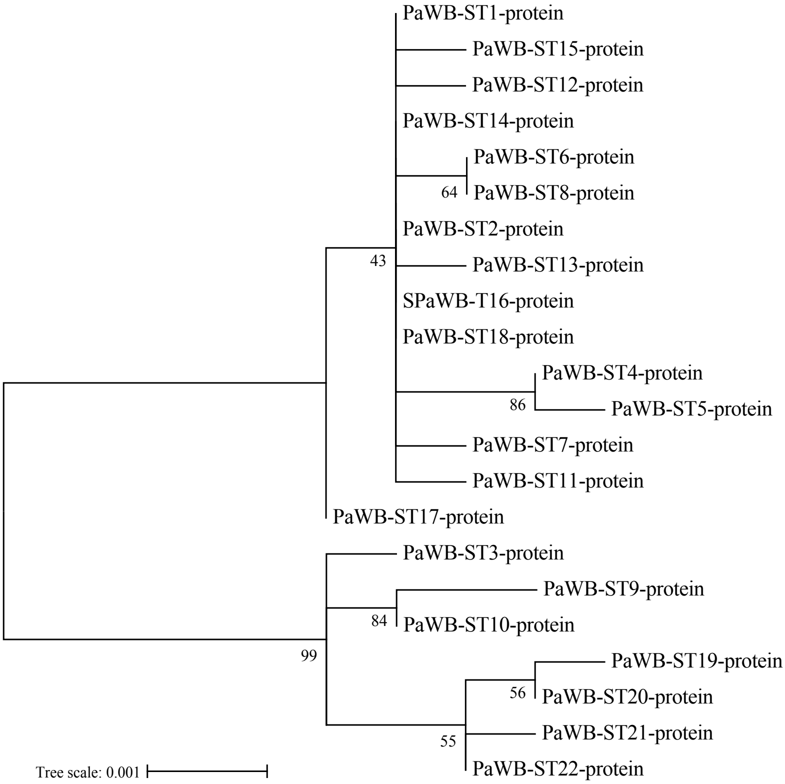

Figure S2. Maximum-likelihood phylogenetic tree for the 22 STs of PaWB phytoplasmas based on the concatenated sequences of six proteins (except ribosomal protein). The scale bar length represents inferred character-state changes under the best-fitting LG+G+I model.

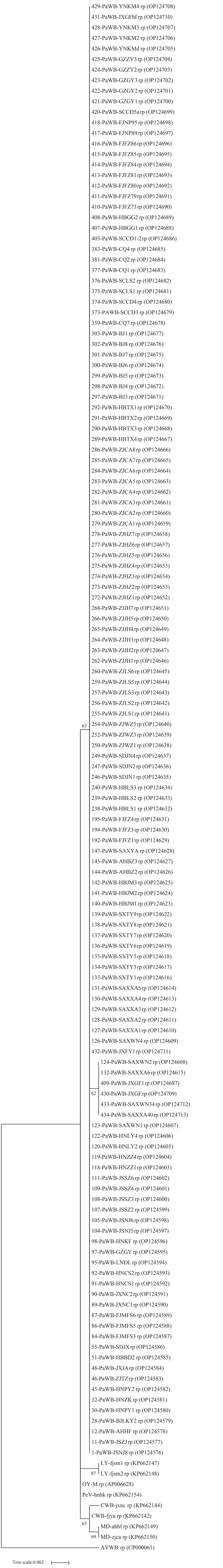

Figure S3. Phylogenetic tree constructed based on the *rp* gene of the PaWB phytoplasma strains and 6 kinds of phytoplasma reference strains employing the maximum-likelihood method.

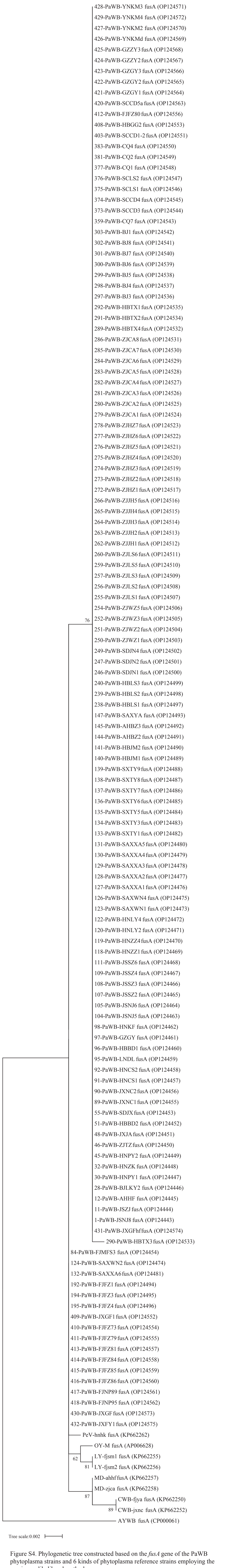

Figure S4. Phylogenetic tree constructed based on the *fusA* gene of the PaWB phytoplasma strains and 6 kinds of phytoplasma reference strains employing the maximum-likelihood method.

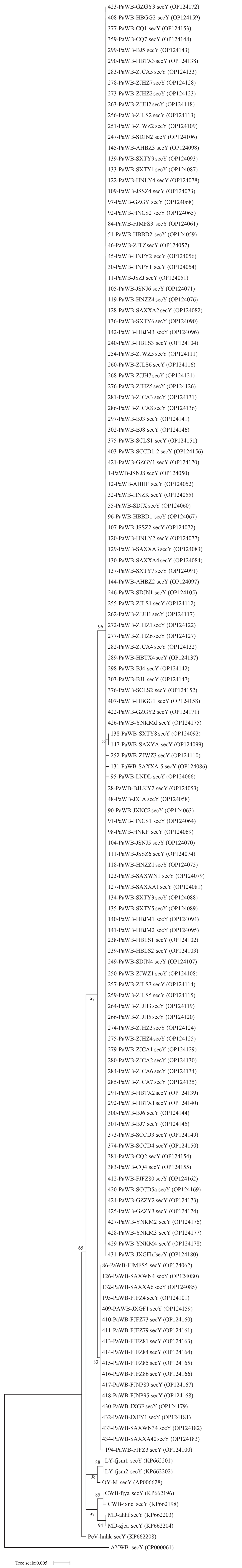

Figure S5. Phylogenetic tree constructed based on the *secY* gene of the PaWB phytoplasma strains and 6 kinds of phytoplasma reference strains employing the maximum-likelihood method.

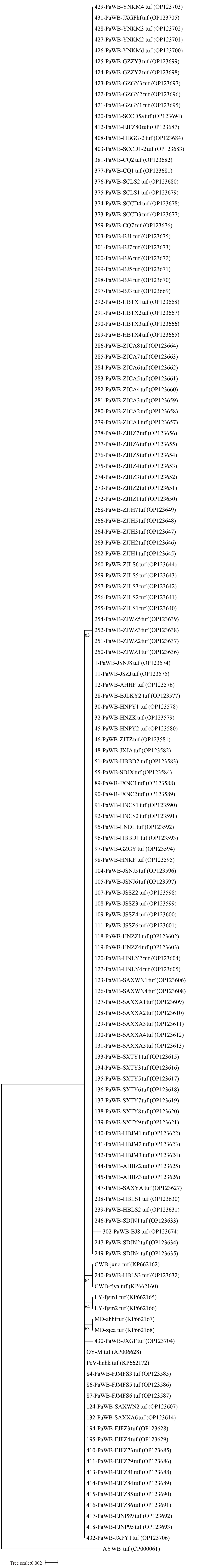

Figure S6. Phylogenetic tree constructed based on the *tuf* gene of the PaWB phytoplasma strains and 6 kinds of phytoplasma reference strains employing the maximum-likelihood method.

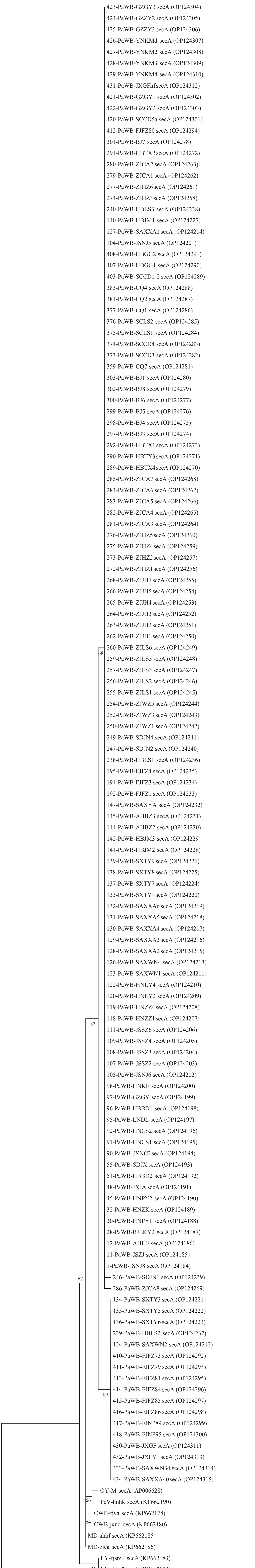

Figure S7. Phylogenetic tree constructed based on the *secA* gene of the PaWB phytoplasma strains and 6 kinds of phytoplasma reference strains employing the maximum-likelihood method.

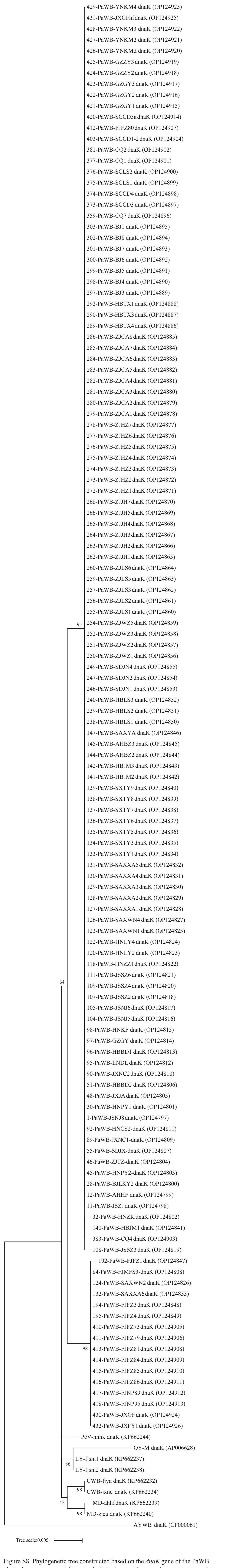

Figure S8. Phylogenetic tree constructed based on the *dnaK* gene of the PaWB phytoplasma strains and 6 kinds of phytoplasma reference strains employing the maximum-likelihood method.

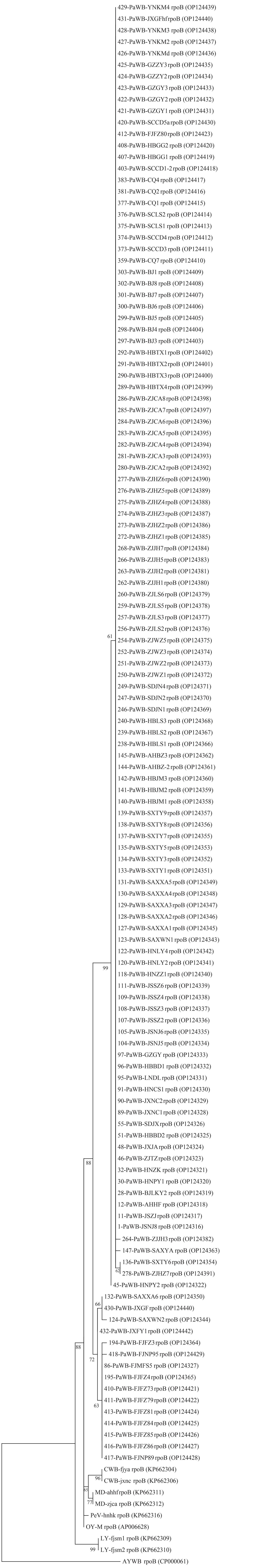

Figure S9. Phylogenetic tree constructed based on the *rpoB* gene of the PaWB phytoplasma strains and 6 kinds of phytoplasma reference strains employing the maximum-likelihood method.

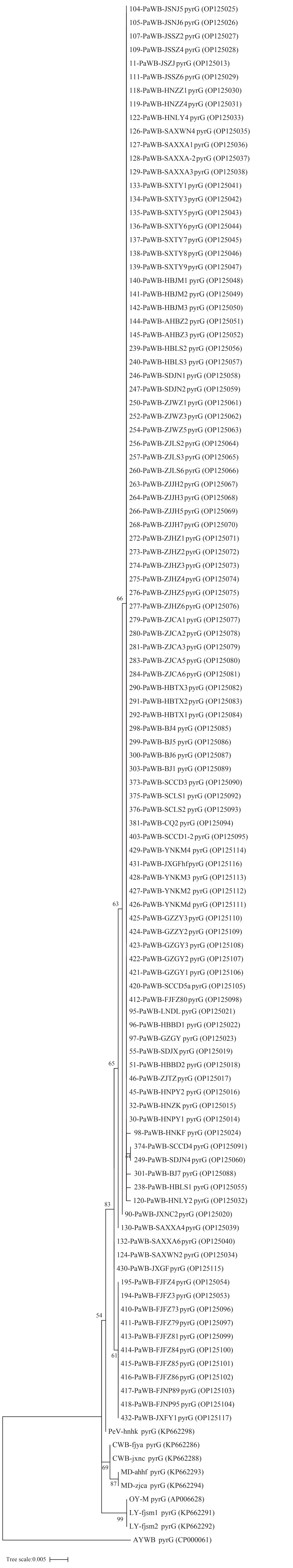

Figure S10. Phylogenetic tree constructed based on the *pyrG* gene of the PaWB phytoplasma strains and 6 kinds of phytoplasma reference strains employing the maximum-likelihood method.

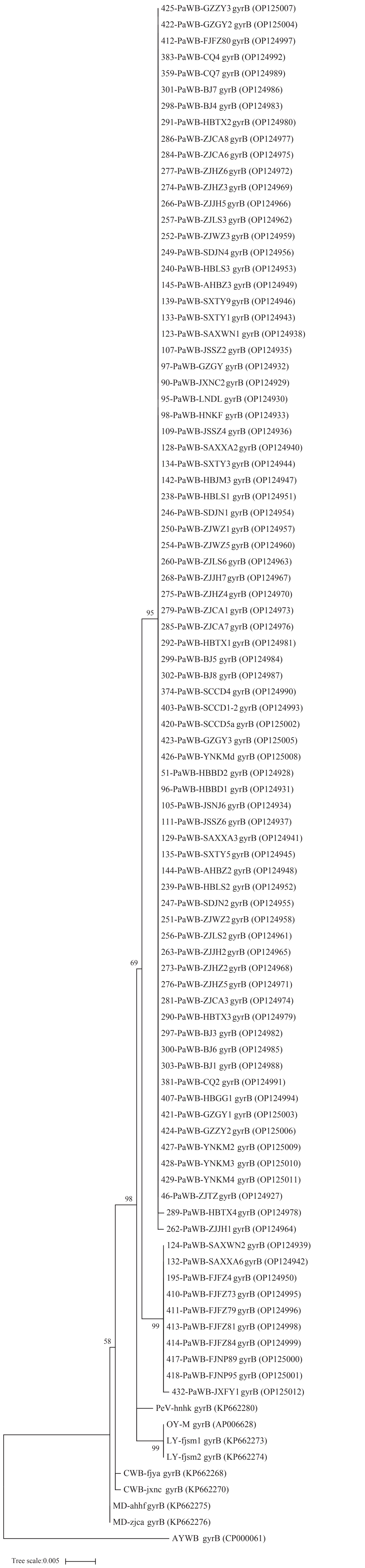

Figure S11. Phylogenetic tree constructed based on the *gyrB* gene of the PaWB phytoplasma strains and 6 kinds of phytoplasma reference strains employing the maximum-likelihood method.

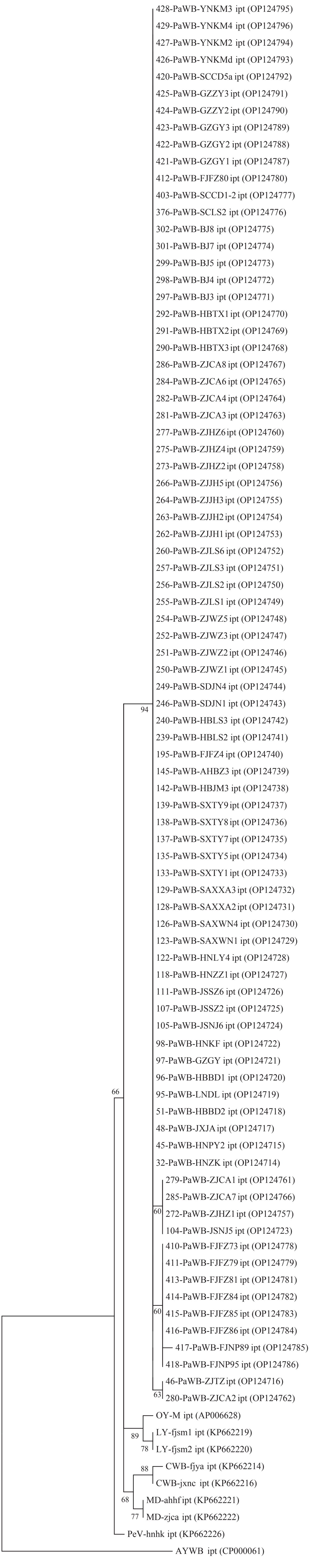

Figure S12. Phylogenetic tree constructed based on the *ipt* gene of the PaWB phytoplasma strains and 6 kinds of phytoplasma reference strains employing the maximum-likelihood method.
